# Pre‐Administration of *Akkermansia Muciniphila* Prevents the Development of Severe Acute Graft‐Versus‐Host Disease in Systemic Organs

**DOI:** 10.1002/advs.202415982

**Published:** 2025-11-30

**Authors:** Jeong‐Eun Han, Dae‐Seong Lee, Su‐Won Jeong, Ji‐Hyun Yun, Seomin Kang, Seoyoung Jang, EunAh Lee, Ju Hye Baek, Che Ok Jeon, Jin‐Woo Bae

**Affiliations:** ^1^ Department of Biology College of Science Kyung Hee University Seoul Republic of Korea; ^2^ Department of Medical Engineering Graduate School Kyung Hee University Seoul Republic of Korea; ^3^ IIRC & Department of Electronic Engineering Kyung Hee University Seoul Republic of Korea; ^4^ Department of Life Science Chung‐Ang University Seoul Republic of Korea

**Keywords:** acute graft‐versus‐host disease, bile acids, gut microbiome, metabolites, prevention effect

## Abstract

Acute graft‐versus‐host disease (aGvHD) remains a significant clinical challenge, with no optimal treatment despite advancements in medical science. This life‐threatening condition, characterized by multi‐organ involvement and high mortality, is increasingly linked to the gut microbiota. Therefore, this study investigates the protective effects of *Akkermansia muciniphila* on aGvHD, focusing on its capacity to modulate the gut microbiome and reduce disease symptoms. *A. muciniphila* is orally administered to mice prior to inducing aGvHD through allogeneic hematopoietic stem cell transplantation. The effects of *A. muciniphila* administration on the development of aGvHD are assessed through disease activity scoring, histological analysis, metabolite and immune profiling, and gut microbiota analyses. Pre‐administration of *A. muciniphila* significantly reduces aGvHD severity, particularly in the gastrointestinal tract, alleviates gut dysbiosis, and increases the levels of metabolites such as tauroursodeoxycholic acid and short‐chain fatty acids. These findings provide the basis for new therapeutic strategies for aGvHD and contribute to improving treatment outcomes for patients with intractable diseases.

## Graphical abstract

*Akkermansia muciniphila*, a next‐generation probiotic, alleviates acute graft‐versus‐host disease (aGvHD) following allogeneic hematopoietic stem cell transplantation (HSCT) by providing protective effects across multiple organs. Pre‐colonization with *A. muciniphila* improves survival and reduces disease severity by stabilizing the gut microbiome, preserving intestinal barrier integrity, restoring bile acid metabolism, and reprogramming disease‐associated transcriptomic and microRNA profiles. *A. muciniphila* enhances protective metabolites such as tauroursodeoxycholic acid (TUDCA) and suppresses intestinal miR‐155 expression, leading to reduced pro‐inflammatory cytokine production, improved immune homeostasis, and maintenance of gut barrier function.

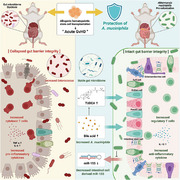

## 1 Introduction

Acute graft‐versus‐host disease (aGvHD) is one of the most challenging and deadly complications of allogeneic hematopoietic stem cell transplantation (HSCT), occurring in ≈30–50% of recipients.^[^ ^1^ ^]^ Patients with aGvHD are characterized by inflammation and activation of cytotoxic immune cells that attack the recipient's tissues, primarily affecting the immune system, gastrointestinal (GI) tract, skin, and mucosa, causing high mortality rates of up to 50%.^[^ ^2^ ^]^

Currently, no effective treatments have been developed for aGvHD; ≈50% of patients do not respond adequately to first‐line steroid therapy and have fatal outcomes.^[^ ^1^ ^]^ Immunosuppressive approaches have been used to prevent the onset of aGvHD.^[^ ^3^, ^4^ ^]^ However, while these methods reduce the incidence of aGvHD, they increase the susceptibility to infectious diseases and tissue damage, ultimately failing to improve overall survival rates.^[^ ^5^, ^6^, ^7^ ^]^ Therefore, controlling and managing aGvHD remains a significant challenge.

The GI tract is particularly susceptible to severe aGvHD damage, leading to significant morbidity.^[^ ^8^ ^]^ The severity of GI tract damage caused by aGvHD is exacerbated by the loss of Paneth and goblet cells, which are essential for maintaining intestinal barrier function and mucosal integrity.^[^ ^9^, ^10^, ^11^ ^]^ Therefore, protecting the GI tract has often been suggested as an approach for alleviating the symptoms of aGvHD.

Recently, studies have highlighted the critical role of the gut microbiome in treating human diseases, emphasizing the essential interplay between the resident microbiota and immune function.^[^ ^12^, ^13^ ^]^ Studies have revealed intricate relationships between the gut microbiome and aGvHD. Analyses of the gut microbiomes of patients with aGvHD have shown a correlation between increased mortality rates and gut dysbiosis.^[^ ^14^, ^15^, ^16^, ^17^ ^]^ Metabolites produced by the gut microbes influence the development of aGvHD; decreased levels of short‐chain fatty acids (SCFAs) and bile acids are associated with disease progression in patients with aGvHD. Additionally, supplementation with metabolites, such as tauroursodeoxycholic acid (TUDCA), alleviates aGvHD symptoms.^[^ ^18^, ^19^ ^]^

*Akkermansia muciniphila* is a gram‐negative, anaerobic bacterium belonging to the phylum Verrucomicrobiota, which is recognized for its immunomodulatory functions and crucial role in the human GI tract.^[^ ^20^ ^]^ Its diverse functions include promoting the proliferation of Paneth and goblet cells, facilitating epithelial development, enhancing the integrity of tight junctions, and maintaining homeostatic immune responses.^[^ ^21^, ^22^ ^]^ Furthermore, *A. muciniphila* has been associated with an improved response to cancer immunotherapy, alleviation of colorectal cancer, and mitigation of radiation‐ and chemotherapy‐induced colitis.^[^ ^23^, ^24^, ^25^ ^]^ Based on this evidence, *A. muciniphila* has been widely used in human disease therapy research.

Additionally, GvHD is known to be regulated by host mRNAs and microRNAs, linking immune activation with intestinal barrier integrity.^[^ ^26^, ^27^ ^]^ Dysregulated expression of cytokine‐related transcripts, such as TNF‐α, IFN‐γ, and IL‐17, amplifies cytotoxic T cell activity and drives the progression of aGvHD‐associated mucosal injury.^[^ ^28^, ^29^ ^]^ Furthermore, inflammatory microRNAs such as miR‐155 exacerbate intestinal inflammation and barrier injury.^[^ ^26^, ^30^, ^31^, ^32^ ^]^ Moreover, commensal bacteria, including *A. muciniphila*, have been suggested to influence these regulatory networks, thereby reinforcing epithelial integrity through modulation of host miRNA pathways.^[^ ^33^, ^34^ ^]^ Together, these studies highlight microbiota–miRNA interactions as a novel therapeutic axis in aGvHD.

Therefore, this study investigated whether *A. muciniphila* could modulate the onset and severity of aGvHD in murine mouse models. Through integrative profiling of host immune and transcriptomic responses alongside gut microbiota and metabolite analyses, we aimed to uncover mechanistic links between *A. muciniphila* and intestinal immune homeostasis. By validating these observations in human cell‐based systems, this study provides a foundation for developing *A. muciniphila*‐based preventive or therapeutic strategies for aGvHD.

## 2 Results

### 2.1 Administration of *A. Muciniphila* Ameliorated aGvHD

**Figure 1:**
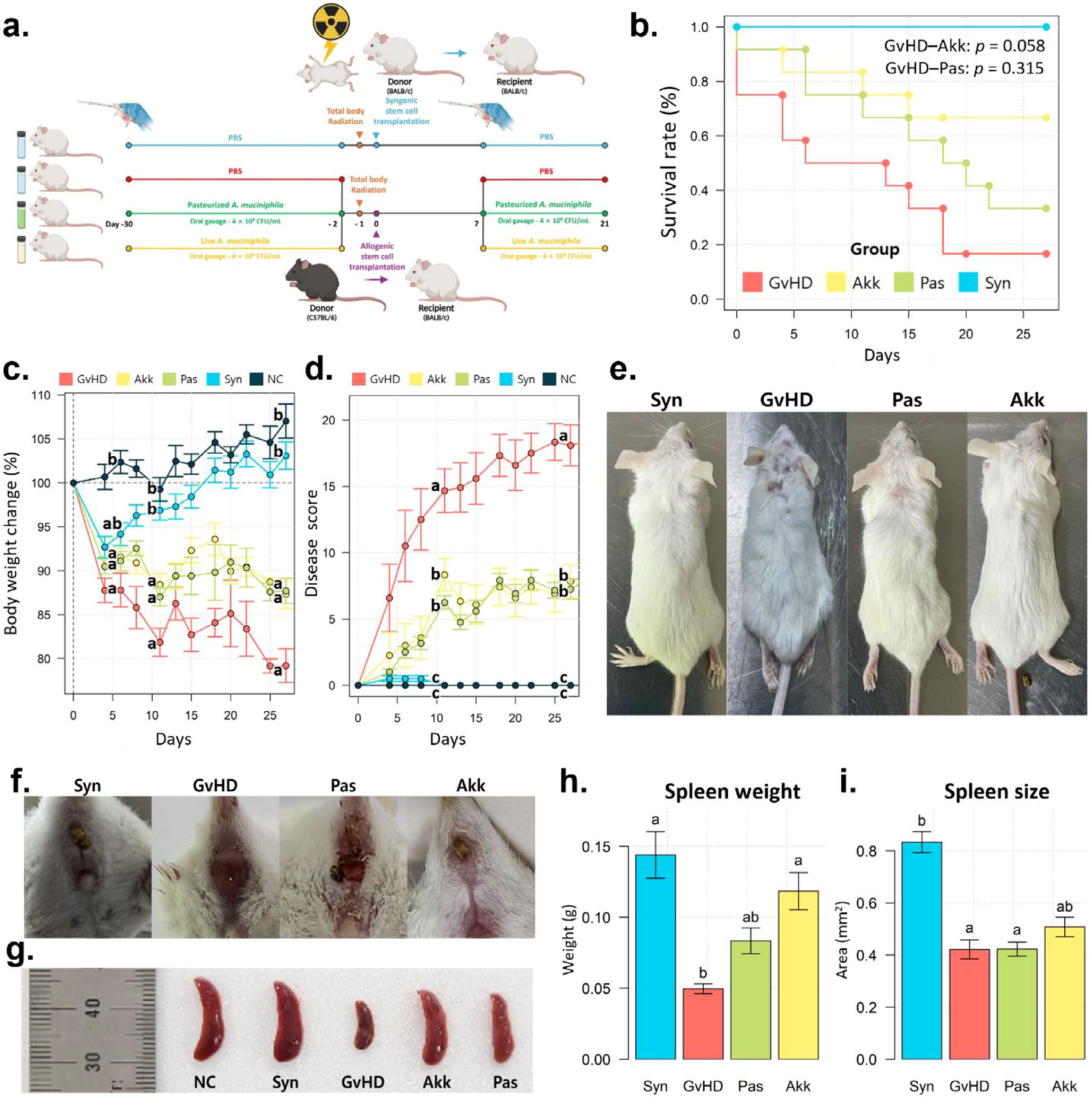
Supplementation with pasteurized or live *A. muciniphila* reduced the severity of aGVHD. a) Schematic design of the experiment. Mice were randomly divided into five groups: GvHD, PBS‐administered aGvHD group; Akk, aGvHD group administered with live *A. muciniphila*; Pas, aGvHD group administered with pasteurized *A. muciniphila*; Syn, PBS‐administered syngeneic‐HSCT group; NC, normal control mice administered only PBS Mice were daily administered PBS, live, or pasteurized *A. muciniphila* by oral gavage from 1 month before HSCT until the onset of disease, and restarted 1 week after disease onset. Recipients were monitored for survival rate b), body weight change c), and clinical score d) up to 30 days after HSCT (n = 5–12). Representative pictures of the overall appearance e) and rectal condition f) in each group were taken. Mice spleens were collected 3 weeks post‐HSCT to confirm the phenotype g), weight h), and size i). Survival analysis with Cox proportional hazards model was used to confirm the difference in survival trends (b) between the Akk and Pas groups for GvHD group. The Kruskal–Wallis test followed by Dunn's multiple comparisons test was performed to determine statistical differences between groups (c, d, h, i). Different letters in the figures indicate statistically significant differences (*p* < 0.05) and error bars indicate the standard error of the mean.

First, we assessed the symptom severity of aGvHD following pre‐administration of live (Akk group) and pasteurized (Pas group) *A. muciniphila* in mice. We administered *A. muciniphila* daily via oral gavage for 1 month prior to HSCT and restarted administration 1 week after disease onset to investigate its potential therapeutic effects (**Figure** **1**a). Syngeneic‐HSCT recipients (Syn group) and normal control mice administered only phosphate‐buffered saline (PBS) were used as healthy control groups (NC group). The PBS‐administered aGvHD (GvHD group) group exhibited severe disease symptoms, including a decreased survival rate (Figure 1b), severe weight loss (Figure 1c), higher clinical scores (Figure 1d), and overall appearance (Figure 1e). In contrast, the Akk group showed significant improvements in aGvHD symptoms, such as a high survival rate and low disease score, compared to those of the GvHD group, although body weight loss did not show a statistically significant difference (*p* = 0.06). The Akk and Pas groups showed notable improvements in GI symptoms due to aGvHD, including a reduced incidence of bloody stools, diarrhea, rectal swelling, and rectal bleeding (Figure 1f). In addition, splenic atrophy was less severe in the Akk and Pas groups than that in the GvHD group (Figure 1g‐i). However, the degree of improvement was greater in the Akk group than that in the Pas group. The preventive trends of both live and pasteurized *A. muciniphila* on aGvHD were observed in other mouse models as well, in which the roles of donor and recipient were reversed (Figure S1, Supporting Information). In addition, administration of *A. muciniphila* after the onset of disease showed a tendency to alleviate disease progression, although the effect was less pronounced than that observed when pre‐ and post‐treatment were combined (Figure S2, Supporting Information).

### 2.2 Pre‐Administration of *A. Muciniphila* Strengthened Gut Barrier and Reduced GI Symptoms Against aGvHD

**Figure 2:**
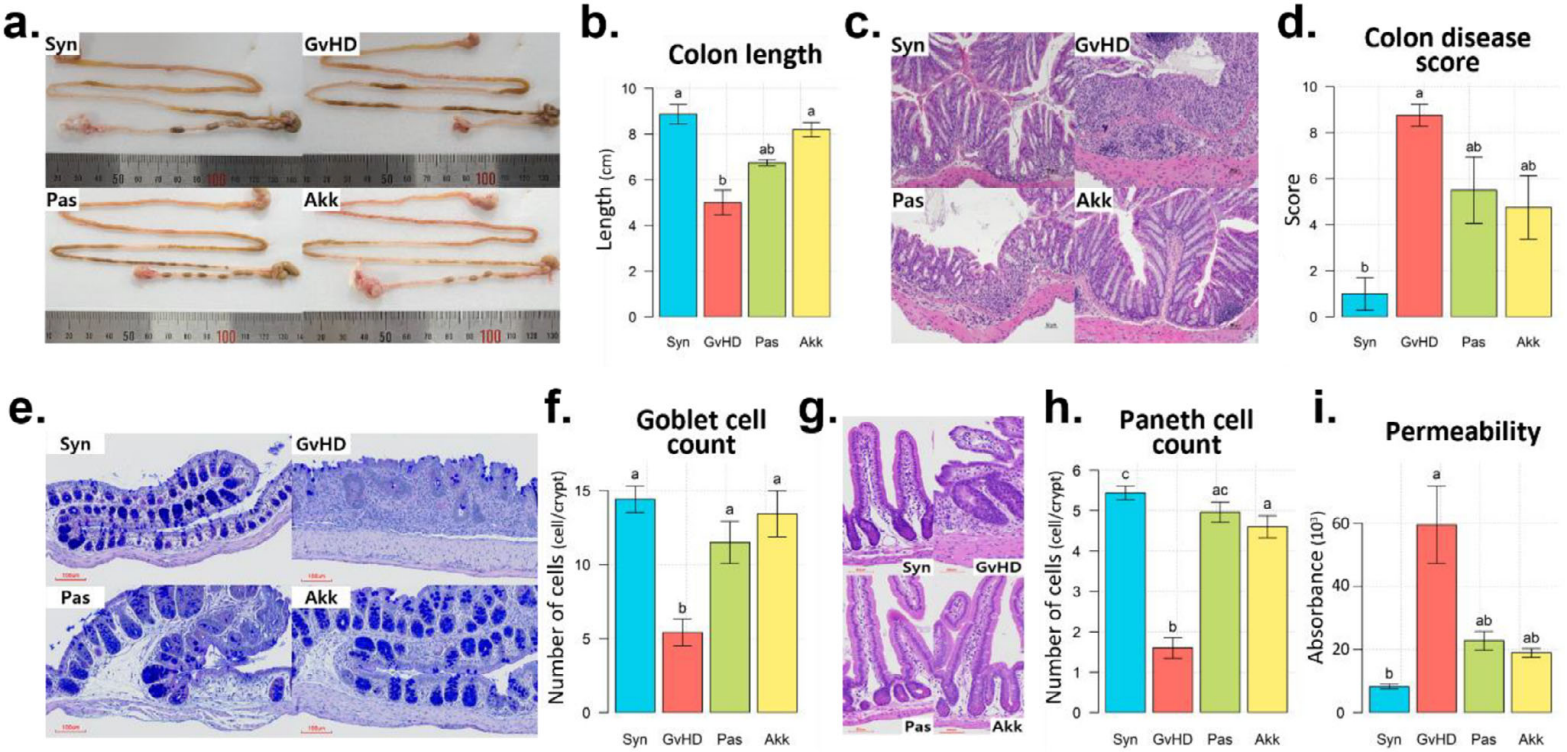
Protective effects of pasteurized or live *A. muciniphila* on GI tract damage caused by aGvHD. The intestinal tracts were collected at 3 weeks post‐HSCT to assess the severity of GI tract damage (n = 3–4 per group). Phenotype images of the GI tract a) and colon length measurements b). Representative histological images of H&E‐stained c) and Alcian blue/PAS‐stained e) colon tissues, along with the corresponding histology scores d) and number of goblet cells per crypt f). Representative H&E‐stained histological images of the small intestine crypts g) and the number of Paneth cells per crypt h). i) Quantification of intestinal permeability by group using the FITC‐dextran assay. The Kruskal–Wallis test followed by Dunn's multiple comparisons test was performed to determine statistical differences between groups (b, d, f, h, i). Different letters in the figures indicate statistically significant differences (*p* < 0.05), and error bars indicate the standard error of the mean. (Mice group abbreviation: GvHD, PBS‐administered aGvHD group; Akk, aGvHD group administered with live *A. muciniphila*; Pas, aGvHD group administered with pasteurized *A. muciniphila*; Syn, PBS‐administered syngeneic‐HSCT group).

The Akk group showed a significantly longer colon than the GvHD group, which was similar to that of the Syn group (**Figure** **2**a,b). Histological examination of the distal colon tissues revealed that the Akk and Pas groups had lower inflammation severity and disease scores than those of the GvHD group (Figure 2c,d). We observed a significant reduction in the number of goblet and Paneth cells per crypt in both cell types in the GvHD group (Figure 2e–h). In contrast, their number was well‐maintained in the Akk and Pas groups, similar to the levels of the Syn group. In addition, the results of the fluorescently‐labeled small molecule (FITC)‐dextran assay showed that intestinal permeability was lower in the Akk and Pas groups than that in the GvHD group (Figure 2i). Consistent with systemic outcomes such as survival, body weight, and spleen weight were not significantly improved when *A. muciniphila* was administered after disease onset (Figure S2, Supporting Information), histological analyses also revealed only a mild tendency toward the preservation of secretory epithelial lineages (Figure S3, Supporting Information). To further explore the pronounced protective effects of pre‐colonization with *A. muciniphila*, we examined whether its ability to mitigate irradiation‐induced intestinal injury could contribute to their benefit. Pre‐administration of live or pasteurized *A. muciniphila* alleviated irradiation‐induced intestinal damage, as evidenced by histological analyses and reduced post‐irradiation diarrhea compared with PBS‐treated controls (Figure S4, Supporting Information).

### 2.3 Systemic Effects of *A. Muciniphila* for Alleviating aGvHD

**Figure 3:**
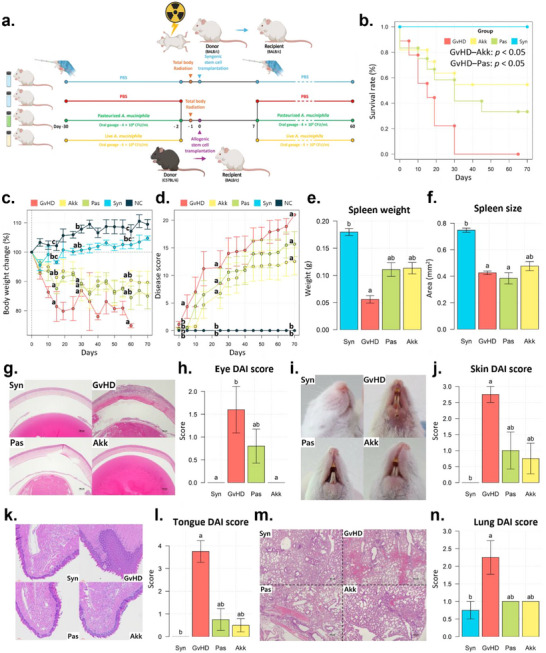
Systemic protective effects of pasteurized or live *A. muciniphila* on aGvHD disease symptoms. a) Schematic of experimental design. Daily gavages of mice with PBS, live, or pasteurized *A. muciniphila* were performed. One month later, a lower dose of HSC was administered. Recipients were monitored for survival rate b), body weight change c), and clinical score d) up to 60 days post HSCT (n = 5–12). e–n) Assessment of disease activity in various tissues at 4 weeks post‐HSCT (n = 3–4 per group). The spleen weight (e) and size (f) were measured for each group. (g) Representative sections of H&E‐stained eyeballs, (h) comparison of disease activity scores for the eye. (i) Typical images of skin phenotype, (j) comparison of disease activity scores for the skin. (k) Representative H&E‐stained histological images of tongue (k) and lung (m) tissues. The disease activity scores shown by bar plots for tongue (l) and lung (n) tissues. Survival analysis with Cox proportional hazards model was used to confirm the difference in survival condition (b) between the Akk and Pas groups for GvHD group. The Kruskal–Wallis test followed by Dunn's multiple comparisons test was performed to determine statistical differences between groups (c–f, h, j, l, n). Different letters in the figures indicate statistically significant differences (*p* < 0.05), and error bars indicate the standard error of the mean. (Mice group abbreviation: GvHD, PBS‐administered aGvHD group; Akk, aGvHD group administered with live *A. muciniphila*; Pas, aGvHD group administered with pasteurized *A. muciniphila*; Syn, PBS‐administered syngeneic‐HSCT group; NC, normal control mice administered only PBS).

In the previous aGvHD model (Figures 1 and 2), the rapid mortality of mice hindered the evaluation of disease symptoms across multiple organs. To slow the mortality, we reduced the proportion of donor T‐cells during the transplantation process (**Figure** **3**a). Similar to previous results, the Akk and Pas groups showed improvements in aGvHD symptoms, resulting in higher survival rates (Figure 3b), lower weight loss (Figure 3c), lower disease scores (Figure 3d), and milder spleen atrophy than those of the GvHD group (Figure 3e,f). Notably, the trend in survival rates between the GvHD and *A. muciniphila* treated groups (Akk and Pas) significantly differed (*p* <0.05). The histopathological scores of the eyes (Figure 3g,h), skin (Figure 3i,j), tongue (Figure 3k,l), and lungs (Figure 3m,n) revealed severe symptoms in the GvHD group, whereas the Akk group showed notably milder symptoms than those of the GvHD group.

### 2.4 Administration of *A. Muciniphila* Alleviated Gut Dysbiosis

**Figure 4:**
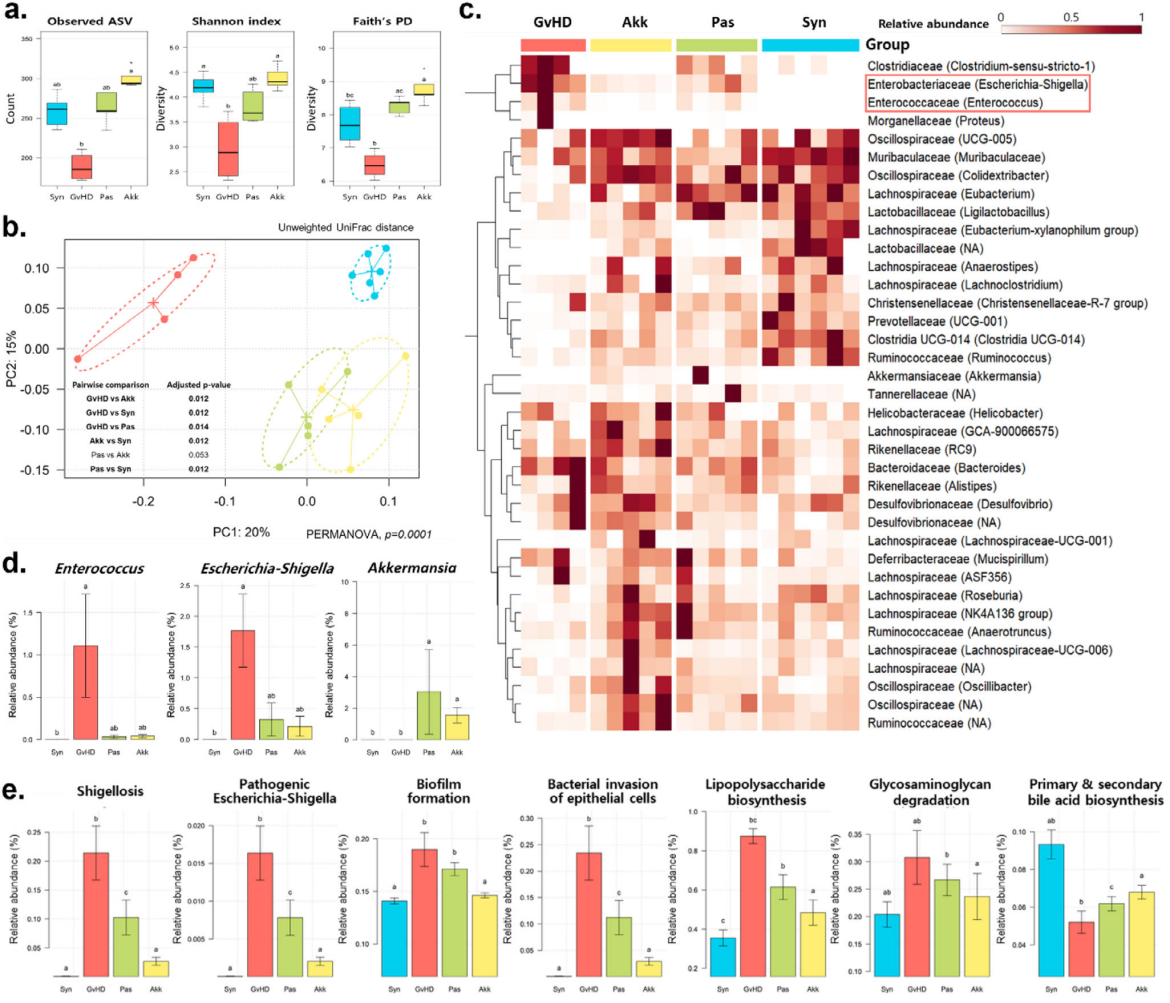
*A. muciniphila*‐induced alleviation is associated with decreased pathogenic bacteria, including *Enterococcus*, and with increased commensal levels. Bacterial 16S rRNA sequencing was performed using fecal samples collected 3 weeks after HSCT (n = 4–6 per group). a) Alpha diversity comparison of each group was performed using observed ASV, Shannon index, and Faith's Phylogenetic Diversity (PD) index. b) Clustering of gut microbiota using PCoA with unweighted UniFrac distances. PERMANOVA was used to determine statistical significance among the groups. c) Relative abundance indicated by a heat map at the genus level. d) Relative abundance of *A. muciniphila*, *Enterococcus*, and *Escherichia*‐*Shigella* by groups e) Relative abundance of predicted functional pathways identified by PICRUSt2 analysis. The Kruskal–Wallis test followed by Dunn's multiple comparisons test was performed to determine statistical differences between groups (a, d, e). Different letters in the figures indicate statistically significant differences (*p* < 0.05), and error bars indicate the standard error of the mean. (Mice group abbreviation: GvHD, PBS‐administered aGvHD group; Akk, aGvHD group administered with live *A. muciniphila*; Pas, aGvHD group administered with pasteurized *A. muciniphila*; Syn, PBS‐administered syngeneic‐HSCT group).

The results of 16S rRNA sequencing of fecal samples showed that the diversity indices of the gut microbial community were significantly decreased in the GvHD group, whereas they were high in the Akk and Pas groups, similar to the Syn group (**Figure** **4**a). According to the principal coordinate analysis (PCoA) results, *A. muciniphila*‐treated (Akk and Pas) and GvHD groups formed distinct clusters representing different microbial communities (Figure 4b). The onset of aGvHD led to a reduction in the number of genera belonging to the Lachnospiraceae and Oscillospiraceae families; however, these increased in the Akk group. In contrast, the GvHD group showed increased levels of *Enterococcus* and *Escherichia‐Shigella*, whereas these levels decreased following *A. muciniphila* treatment (Akk and Pas groups) (Figure 4c,d). These microbial community alterations due to aGvHD were also observed in short read archive (SRA) data from healthy individuals and patients with aGvHD (Figure S5, Supporting Information). Particularly, *Enterococcus* was significantly enriched in aGvHD patients (*p*<0.001), while *A. muciniphila* markedly reduced its levels (*p*<0.001). *Escherichia‐Shigella* did not show a significant difference (*p* = 0.505). Among the gut microbial functional pathways, the GvHD group showed high abundance in infectious disease pathways, including Shigellosis, Escherichia infection, Vibrio biofilm formation, and epithelial cell bacterial invasion, such as the lipopolysaccharide biosynthesis pathway accelerating host inflammation and glycosaminoglycan degradation, which decrease the mucosal layer. However, their levels were notably reduced in the Akk and Pas groups (Figure 4e). To further investigate the mechanism underlying pathogen suppression, we examined whether fecal and intestinal protein extracts from the Syn, Akk, and Pas groups inhibited the growth of *Enterococcus* and *Escherichia–Shigella* compared with those from the GvHD group. Growth of both pathogens was significantly suppressed by extracts from the Syn, Akk, and Pas groups, whereas no inhibitory effect was observed with those from the GvHD group (Figure S6a–b, Supporting Information). Consistently, Reg3γ and lysozyme expression, which were decreased in the GvHD group, were preserved in the Akk and Pas groups, as shown by immunohistochemistry and ELISA (Figure S6c–f, Supporting Information). In contrast to pre‐colonization, administration of *A. muciniphila* after disease onset resulted in only modest alterations in the gut microbiota (Figure S7, Supporting Information).

### 2.5 Increase in Bile Acid Biosynthesis and Metabolites Changes Induced by *A. Muciniphila*

**Figure 5:**
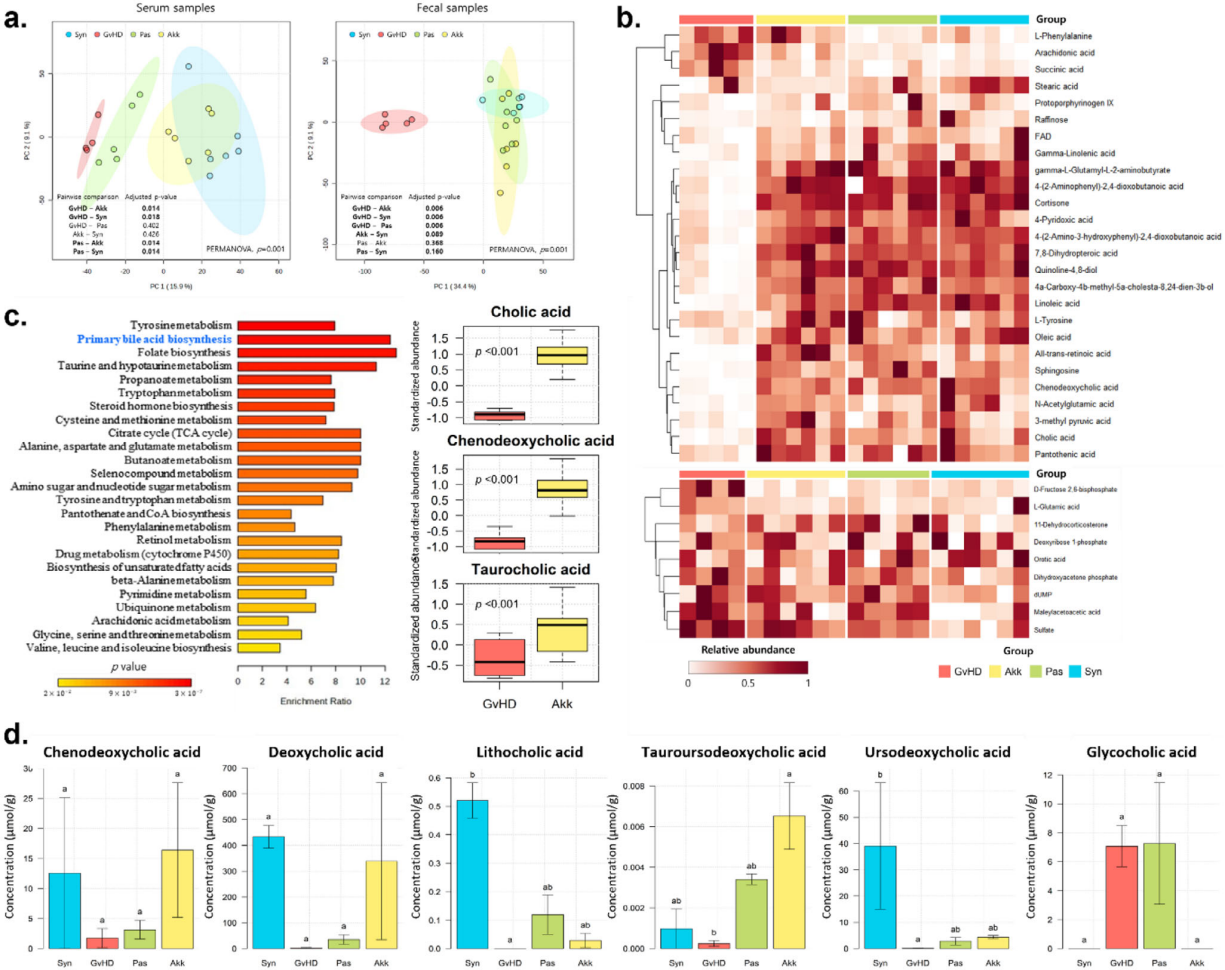
Metabolites alterations associated with *A. muciniphila*‐induced aGvHD alleviation. Untargeted metabolomic analyses were conducted using fecal and serum samples (n = 4–6 per group). a) Principal component analysis (PCA) plots of metabolites from serum (left) and fecal (right) samples. b) Heatmap showing fecal (top) and serum (bottom) compounds with significant differences by group. c) KEGG pathway enrichment analysis of differentially enriched fecal metabolites between the GvHD and Akk groups. Statistical analysis using the Wilcoxon rank‐sum test was performed to identify significant differences in bile acid levels between the GvHD and Akk groups. d) Targeted metabolomic analysis of bile acids using fecal samples (n = 3). The Kruskal–Wallis test followed by Dunn's multiple comparisons test was performed to determine statistical differences between groups. Different letters in the figures indicate statistically significant differences (*p* < 0.05), and error bars indicate the standard error of the mean. (Mice group abbreviation: GvHD, PBS‐administered aGvHD group; Akk, aGvHD group administered with live *A. muciniphila*; Pas, aGvHD group administered with pasteurized *A. muciniphila*; Syn, PBS‐administered syngeneic‐HSCT group).

Untargeted metabolomic analysis of fecal and serum samples collected from aGvHD mice revealed distinct clusters among the Akk, Syn, and GvHD groups (**Figure** **5**a). The GvHD group showed a marked increase in metabolites related to pro‐inflammatory characteristics, such as arachidonic acid, succinic acid, and l‐phenylalanine (Figure 5b). However, these values were lower in Akk and Pas groups. Conversely, metabolites associated with anti‐inflammatory properties and gut barrier protective effects, such as L‐tyrosine, oleic acid, pantothenic acid, and retinoic acid, were decreased in the GvHD group, whereas their levels were preserved in the Akk group. Consistent with this, short‐chain fatty acid (SCFA) levels, including butyrate, acetate, and propionate were decreased in the GvHD group but were partially restored in the Akk and Pas groups (Figure S8, Supporting Information).

According to enrichment analysis between Akk and GvHD groups based on the Kyoto Encyclopedia of Genes and Genomes (KEGG) database, bile acid biosynthesis pathway was significantly altered (Figure 5c). In addition, PICRUSt2 analysis revealed that the bile acid biosynthesis pathway significantly decreased in the GvHD group and increased in the Akk and Pas groups (Figure 4e). Targeted metabolomic analysis of fecal samples revealed that the levels of chenodeoxycholic acid, deoxycholic acid, lithocholic acid, TUDCA, and ursodeoxycholic acid decreased in the GvHD group but increased in the Akk group. Among them, TUDCA showed a statistically significant increase (Figure 5d). In line with these changes, analysis of predicted functional pathways showed enrichment of taurine‐related pathways in the Akk group compared with the Pas group (Figure S9, Supporting Information).

To further determine whether the preventive effects of live and pasteurized *A. muciniphila* were linked to metabolic changes before disease onset, we performed metabolite profiling. The analysis revealed that administration of live *A. muciniphila* increased TUDCA levels compared with the PBS‐ or pasteurized *A. muciniphila*–treated groups (Figure S10, Supporting Information). Since metabolomic analyses revealed an increase of TUDCA levels in Akk group during aGvHD, we further conducted in vitro assays using human Peripheral blood mononuclear cells (PBMCs) and HT‐29 epithelial cells. Inflammatory PBMCs decreased HT‐29 cell viability and increased epithelial permeability, whereas co‐treatment with TUDCA reduced these effects. Notably, the protective action of TUDCA was abolished by its antagonist SBI‐115 (Figure S11, Supporting Information).

### 2.6 Amelioration of Immune Dysregulation by *A. Muciniphila*

**Figure 6:**
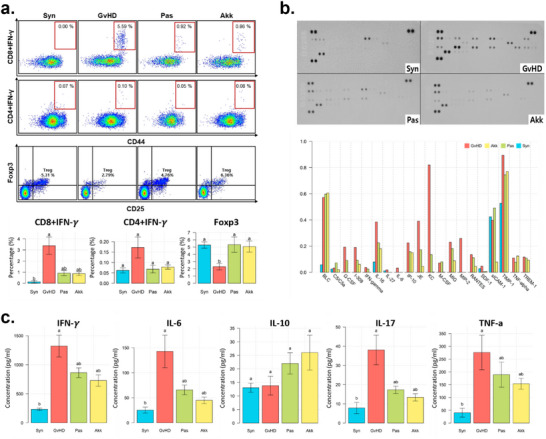
Supplementation with live or pasteurized *A. muciniphila* mitigates severe aGvHD by influencing immune response and hematologic function. a) Peripheral blood from recipients was collected 3 weeks post‐HSCT and analyzed via flow cytometry for IFN‐γ and Foxp3 expression. Data presented include the representative images (up) and corresponding bar plots (down). b) Proteomic analysis of inflammatory chemokines and chemokines using serum samples by each group. c) Quantification of inflammatory cytokines using ELISA. Serum samples were collected from each group and analyzed for the levels of IFN‐γ, IL‐6, IL‐17, TNF‐α, and IL‐10. The Kruskal–Wallis test followed by Dunn's multiple comparisons test was performed to determine statistical differences between groups (a, c). Different letters in the figures indicate statistically significant differences (*p* < 0.05) and error bars indicate the standard error of the mean. (Mice group abbreviation: GvHD, PBS‐administered aGvHD group; Akk, aGvHD group administered with live *A. muciniphila*; Pas, aGvHD group administered with pasteurized *A. muciniphila*; Syn, PBS‐administered syngeneic‐HSCT group).

According to flow cytometry analysis using PBMCs (**Figure** **6**a), the proportion of IFN‐γ‐positive T cells, a marker of T‐cell activation, increased in the GvHD group and decreased in the Akk and Pas groups. In contrast, the proportion of Foxp3+ T cells that reduced excessive inflammatory responses was significantly suppressed in the GvHD group and increased in the Akk and Pas groups. Similar to IFN‐γ‐positive T cells, proteome‐based array revealed that chemokines and cytokines, such as IL‐16, CCL2, CXCL10, CXCL9, CCL5, and CXCL2, involving T‐cell recruitment and inflammatory responses, increased in the GvHD group (Figure 6b). Through ELISA, we further confirmed that pro‐inflammatory cytokines, including IFN‐γ, IL‐6, IL‐17, and TNF‐α, were markedly increased in the GvHD group, whereas they were reduced in the Akk and Pas groups (Figure 6c). Conversely, IL‐10, a cytokine that mitigates inflammation, showed the highest levels in the Akk group.

### 2.7 Barrier‐Protective Interventions by *A. Muciniphila* Improve Hematopoietic Function Under Inflammatory Stress

**Figure 7:**
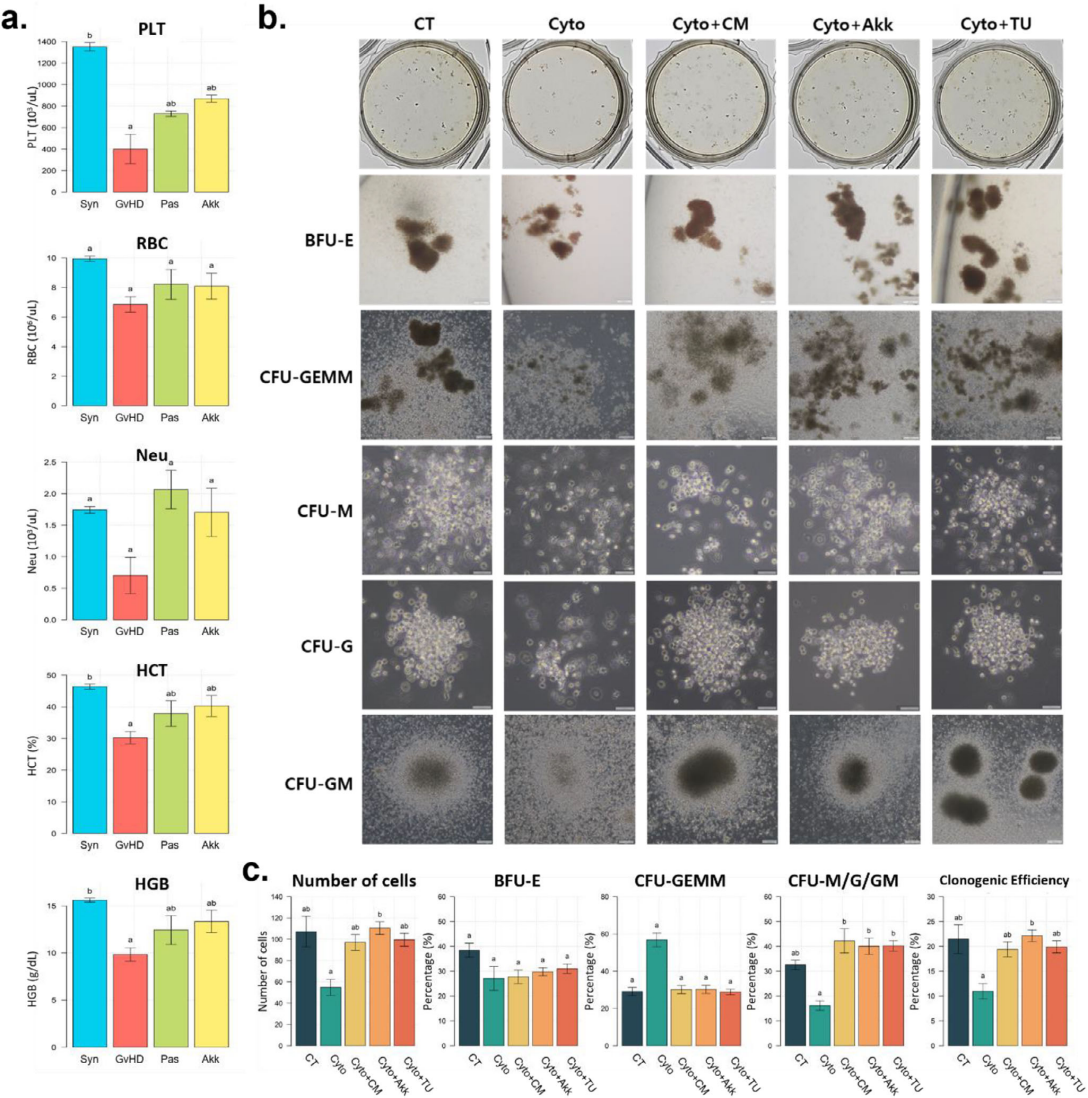
Barrier‐protective treatments with *A. muciniphila* and TUDCA alleviate inflammation‐induced hematopoietic suppression. a) Complete blood count analysis of peripheral blood samples showing platelet (PLT), red blood cell (RBC), neutrophil (Neu), hematocrit (HCT), and hemoglobin (HGB) levels in each group. b) Representative colony‐forming unit (CFU) assay images showing the differentiation of human CD34⁺ hematopoietic stem/progenitor cells (HSPCs) co‐cultured with intestinal epithelial HT‐29 cells under cytokine‐induced inflammatory conditions. Colonies of erythroid burst‐forming units (BFU‐E), granulocyte/erythroid/monocyte/megakaryocyte progenitors (CFU‐GEMM), macrophage progenitors (CFU‐M), granulocyte progenitors (CFU‐G), and granulocyte/macrophage progenitors (CFU‐GM) are shown. c) Total colony numbers and clonogenic efficiency derived from the same CFU assays, along with quantitative analyses of CFU subtypes (BFU‐E, CFU‐GEMM, and CFU‐G/M/GM), were compared across treatment groups. The Kruskal–Wallis test followed by Dunn's multiple comparisons test was performed to determine statistical differences between groups. Different letters in the figures indicate statistically significant differences (*p* < 0.05), and error bars indicate the standard error of the mean. (Abbreviation in Mice groups (a): GvHD, PBS‐administered aGvHD group; Akk, aGvHD group administered with live *A. muciniphila*; Pas, aGvHD group administered with pasteurized *A. muciniphila*; Syn, PBS‐administered syngeneic‐HSCT group. Abbreviation in In vitro treatment groups (b,c): CT, untreated control; Cyto, cytokine‐challenged group (IFN‐γ + TNF‐α + LPS); Cyto+CM, cytokine + *A. muciniphila*–conditioned medium; Cyto+Akk, cytokine + pasteurized *A. muciniphila*; Cyto+TU, cytokine + TUDCA).

Moreover, hematological analysis showed that platelet and neutrophil counts were dramatically reduced in the GvHD group but increased in the Akk and Pas groups (**Figure** **7**a). To further investigate the mechanism, we used a transwell co‐culture model of the gut–hematopoiesis axis (Figure 7b,c). Severe inflammatory stimulation markedly reduced total colony numbers, with pronounced suppression of lineage‐committed progenitors, including granulocyte/macrophage (CFU‐G/M/GM) colonies, whereas the primitive multipotent compartment (CFU‐GEMM) was minimally affected compared with untreated controls (CT). In contrast, treatments with *A. muciniphila*, TUDCA, or *A. muciniphila*‐conditioned medium (CM) significantly improved colony formation at the CFU‐M/G/GM subtypes. Moreover, treatment with *A. muciniphila* significantly enhanced myeloid clonogenic efficiency (Figure 7c). Consistent with these quantitative findings, morphological observation revealed that colonies under *A. muciniphila* and TUDCA treatment were larger, denser, and more mature than those in the cytokine group (Figure 7b).

### 2.8 RNA‐Seq and Microrna‐Seq Revealed Suppression of GvHD Pathway and Regulation of miR‐155 by *A. Muciniphila*

**Figure 8:**
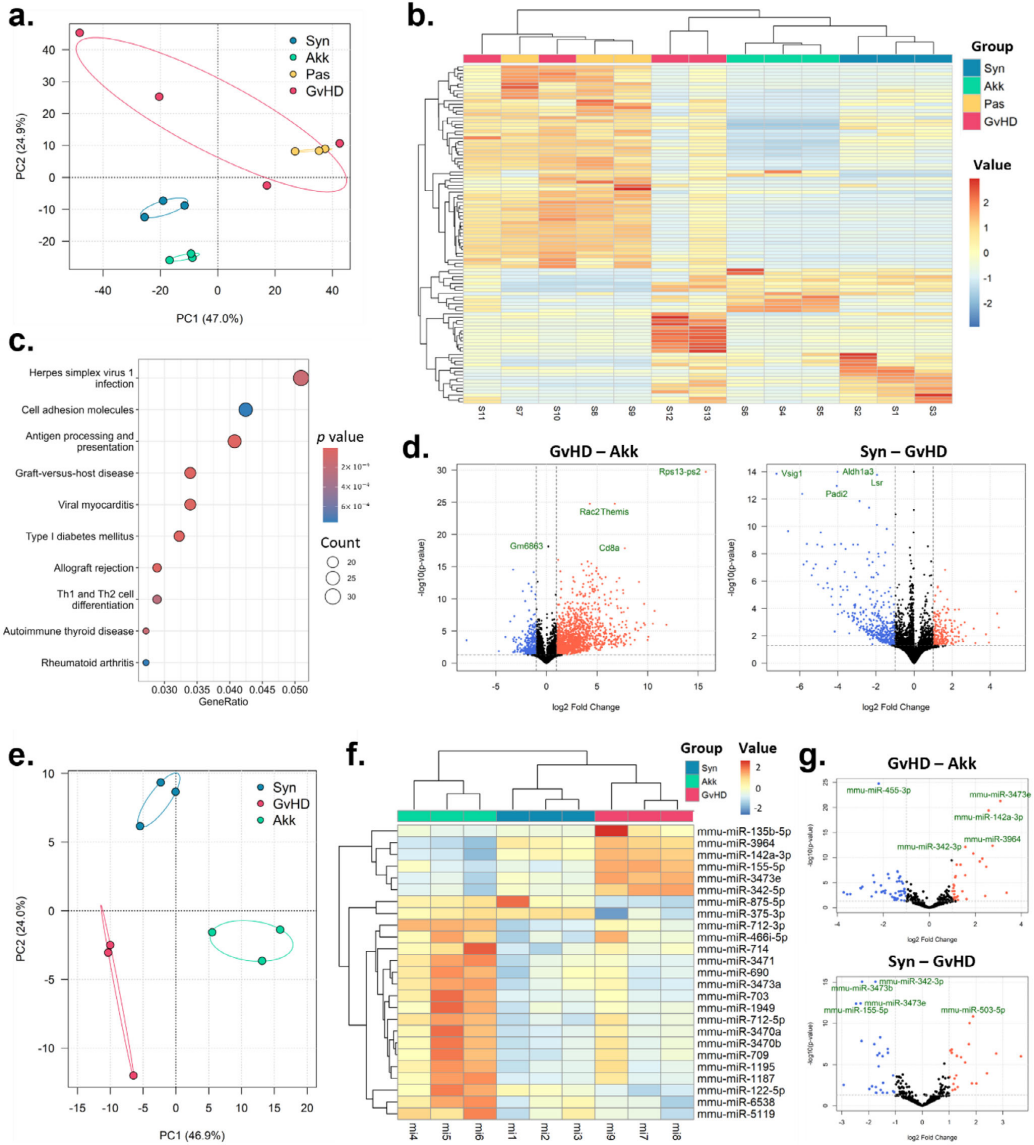
Transcriptomic and microRNA alterations associated with *A. muciniphila*‐induced aGvHD alleviation. RNA‐seq a–d) was performed on intestinal tissues from the Syn, Akk, Pas, and GvHD groups (n = 3–4 per group), and miRNA‐seq e–g) was performed on samples from the Syn, Akk, and GvHD groups. (a) Principal component analysis (PCA) showing global separation among the four groups. (b) Heatmap of differentially expressed genes (DEGs) across the four groups. (c) KEGG over‐representation analysis of DEGs comparing GvHD versus Akk groups (downregulated pathways). (d) Volcano plots for GvHD versus Akk and Syn versus GvHD highlighting the top significantly altered genes. (e) PCA based on miRNA expression showing clear separation among the three groups. (f) Heatmap of differentially expressed miRNAs across the three groups. (g) Volcano plots comparing GvHD versus Akk and Syn versus GvHD, highlighting significantly altered miRNAs (including miR‐155 where applicable). Significance for DEG analyses was defined as adjusted *p* < 0.05 with |log_2_ fold change| > 1; in volcano plots, red and blue denote up‐ and down‐regulated features, respectively. (Mice group abbreviation: GvHD, PBS‐administered aGvHD group; Akk, aGvHD group administered with live *A. muciniphila*; Pas, aGvHD group administered with pasteurized *A. muciniphila*; Syn, PBS‐administered syngeneic‐HSCT group).

RNA‐seq showed distinct clustering in the experimental groups, with the Akk separated from the GvHD group as shown by principal component analysis (PCA). Heatmap visualization further confirmed differential expression patterns among conditions (**Figure** **8**a,b). Notably, KEGG over‐representation analysis (ORA) revealed that immune‐related pathways, including GvHD signaling, were significantly upregulated in the GvHD group, whereas these signals were markedly reduced following treatment of *A. muciniphila* (Figure 8c). Differentially expressed genes (DEGs) were further visualized using volcano plots, which showed marked transcriptional alterations between GvHD and Akk groups, as well as between GvHD and Syn groups (Figure 8d). Comparison between live and pasteurized *A. muciniphila* further revealed that the Akk group exhibited broader downregulation of antigen presentation and GvHD–related pathways than Pas group (Figure S12, Supporting Information).

Given that disease improvement was more pronounced in the Akk group than in the Pas group and that RNA‐seq showed no distinct separation between Pas and GvHD samples (Figure 8a,b), we focused on comparing Syn, Akk and GvHD groups through microRNA‐seq to further investigate the underlying mechanisms. A clear separation between groups was observed, and heatmap analysis identified multiple microRNAs that were altered in response to disease and treatment with *A. muciniphila* (Figure 8e,f). Among these, miR‐155 was markedly upregulated in the GvHD group compared with Syn group, whereas administration of *A. muciniphila* reduced its expression, as further supported by the volcano plot analysis (Figure 8f,g). The role of miR‐155 in epithelial barrier regulation was confirmed using human PBMCs and HT‐29 cells. Transfection with a miR‐155 mimic aggravated epithelial injury, whereas treatment with pasteurized *A. muciniphila* or TUDCA alleviated the loss of cell viability and barrier function (Figure S13, Supporting Information).

## 3 Discussion

Despite notable medical advances, the optimal treatment for aGvHD has not been established. As a severe complication of HSCT, aGvHD is still associated with substantial morbidity and mortality, highlighting the need for improved preventive and therapeutic approaches. Because the disease affects multiple organs and involves both immune and epithelial injury, strategies that provide systemic protection while maintaining intestinal homeostasis are particularly desirable.^[^ ^2^, ^7^, ^11^, ^35^, ^36^ ^]^ *A. muciniphila* has been recognized for its ability to strengthen the intestinal barrier^[^ ^33^, ^37^, ^38^ ^]^ and modulate immune responses,^[^ ^20^, ^33^, ^39^ ^]^ suggesting potential benefits in aGvHD.

aGvHD is characterized by intestinal dysbiosis and pathogenic expansion, particularly of *Enterococcus*, which aggravates disease severity^[^ ^15^, ^43^, ^44^ ^]^ and has been associated with Paneth cell depletion and loss of epithelial antimicrobial defense in patients.^[^ ^11^, ^45^ ^]^ *A. muciniphila* mitigated these effects by maintaining epithelial barrier integrity and preserving Paneth cells. This preservation sustained the secretion of antimicrobial molecules such as Reg3γ and lysozyme. By maintaining barrier function and limiting dysbiosis, *A. muciniphila* prevented pathogenic expansion, including *Enterococcus* and *Escherichia–Shigella*, ultimately alleviating intestinal injury.

Although several studies have explored the relationship between *A. muciniphila* and aGvHD, their findings remain inconsistent. Shono et al.^[^ ^40^ ^]^ reported that *A. muciniphila*, which is increased with antibiotic use in patients with aGvHD, might degrade the mucus layer, thereby potentially exacerbating aGvHD. Yang et al.^[^ ^41^ ^]^ showed that stearic acid, a biomarker of aGvHD, leads to an increase in *A. muciniphila* and worsens the disease. Conversely, Ilett et all.^[^ ^15^ ^]^ found that patients with aGvHD had lower levels of *A. muciniphila*. Aggarwal et al.^[^ ^42^ ^]^ showed that administration of osteopontin mitigated the symptoms of aGvHD and caused an increase in *A. muciniphila*. Additionally*, A. muciniphila* reduces inflammation and promotes gut health in stressful situations, such as radiation‐induced colitis.^[^ ^37^, ^38^ ^]^ These findings suggest that further studies are required to elucidate the role of this bacterium in aGvHD.

The gut microbiome‐derived bile acid biosynthesis pathway plays a critical role in protecting against GvHD, as reduced microbial bile acid metabolism has been shown to exacerbate T cell–mediated inflammation.^[^ ^46^ ^]^ Metabolomic profiling further supported that bile acid biosynthesis decreased during aGvHD development and that it was increased by *A. muciniphila*. Notably, *A. muciniphila* significantly increased TUDCA levels. TUDCA has been reported to mitigate aGvHD by protecting the GI tract from cytokine‐induced apoptosis, reducing antigen presentation, and preserving the graft‐versus‐leukemia effect.^[^ ^19^ ^]^

Moreover, pathway analysis revealed enrichment of taurine‐related metabolism, which may contribute to the elevated TUDCA levels observed in the live *A. muciniphila* group compared with the pasteurized group. Consistent with these metabolic changes, live *A. muciniphila* induced broader suppression of antigen presentation and GvHD‐related pathways than the pasteurized form, indicating greater immunoregulatory potential. This effect, together with elevated TUDCA levels, suggests a potential involvement of the TGR5–cAMP–IL‐10 signaling axis, which has been reported to mediate bile acid–induced anti‐inflammatory responses.^[^ ^47^, ^48^ ^]^

Given that bile acid signaling directly modulates immune activation, these metabolic alterations may explain the immunoregulatory changes observed in our study. Herein, *A. muciniphila* decreased the inflammatory immune profile, including activated cytotoxic T cells and inflammatory cytokines, while significantly increasing the levels of the anti‐inflammatory cytokine IL‐10 and the proportion of Foxp3‐positive T cells. *A. muciniphila*‐derived substrates, such as AmTARS and Amuc_1100, have been reported to increase IL‐10 levels and decrease inflammatory cytokines, such as IL‐6 and TNF‐α.^[^ ^38^, ^39^ ^]^ These findings support the view that *A. muciniphila* attenuates aGvHD by modulating immune responses.

Our transcriptome and microRNA analyses revealed that *A. muciniphila* suppressed GvHD‐related signaling pathways and modulated key miRNA, supporting the role of inflammation in GvHD. At the transcriptome level, RNA‐seq analysis demonstrated a marked downregulation of the GvHD pathway in the Akk group compared with the GvHD group, consistent with the overall attenuation of disease symptoms. This broad downregulation of GvHD‐associated inflammatory pathways indicates that *A. muciniphila* exerts systemic protection that extends beyond the regulation of individual cytokines or immune cell types.

In particular, the expression of miR‐155, a well‐established driver that promotes the development of inflammatory T cells,^[^ ^30^ ^]^ was significantly reduced in Akk group. Notably, miR‐155 has been reported to be upregulated in the intestinal tissues of patients with aGvHD, where it amplifies inflammatory signaling.^[^ ^26^, ^49^ ^]^ Thus, the downregulation of miR‐155 in *A. muciniphila*‐treated mice provides mechanistic support for its intestinal protective role. A recent study further demonstrated that *A. muciniphila*–derived vesicles downregulate miR‐155 and enhance IL‐10 in human dendritic cells,^[^ ^50^ ^]^ suggesting that vesicle‐mediated signaling may contribute to the pronounced efficacy observed with live bacteria. Together, these findings indicate that *A. muciniphila* not only reinforces epithelial barrier integrity but also rebalances host miRNA networks, with suppression of miR‐155 providing a mechanistic link to its immunomodulatory effects.

In addition, *A. muciniphila* attenuated hematologic dysfunction, a common complication in patients with aGvHD.^[^ ^51^, ^52^ ^]^ Chronic inflammation is known to impair hematopoietic stem cell (HSC) self‐renewal and accelerate hematopoietic aging,^[^ ^53^ ^]^ Consistent with this, our findings suggest that *A. muciniphila* and its metabolite TUDCA preserve hematopoietic function under inflammatory stress by maintaining intestinal barrier integrity and protecting inflammation‐sensitive progenitor stages. This barrier‐mediated protection aligns with the emerging concept of the gut–bone marrow axis, in which intestinal homeostasis contributes to systemic hematopoietic resilience. Moreover, prior studies have indicated that TUDCA can enhance viability of erythroid stages derived from CD34⁺ HSCs^[^ ^54^ ^]^ and promote progenitor mobilization,^[^ ^55^ ^]^ supporting its role in maintaining hematopoietic potential under stress.

In this study, both preventive and therapeutic administrations of *A. muciniphila* were evaluated. Pre‐administration markedly reduced the development of severe aGvHD. Given that conditioning irradiation aggravates gut injury and triggers inflammation initiating aGvHD,^[^ ^56^ ^]^ the ability of *A. muciniphila* to protect against irradiation‐induced epithelial damage likely accounts for its stronger prophylactic efficacy.

Furthermore, *A. muciniphila* retains beneficial effects even after pasteurization, including enhancement of the intestinal barrier and attenuation of inflammation.^[^ ^23^, ^57^ ^]^ In our study, both live and pasteurized forms alleviated aGvHD symptoms, moreover, the pasteurized form also demonstrated protective effects in human epithelial cell assays. Given that the pasteurized strain lacks mucin‐degrading activity and poses minimal infection risk, it represents a safer and more clinically translatable therapeutic option for immunocompromised HSCT patients.

Herein, we demonstrated the protective effects of *A. muciniphila* against aGvHD. However, the high mortality rate limited the number of mice available for data analysis, including statistical tests. Therefore, future studies with larger sample sizes are warranted to address these quantitative limitations and to further clarify the effects of *A. muciniphila* on aGvHD. In addition, given that the onset of aGvHD is derived from HSCT for hematological malignancies, further studies are needed to determine the possible effects of *A. muciniphila* on patients undergoing HSCT beyond its preventive effects against aGvHD. Moreover, considering that the effects of *A. muciniphila* observed in this study contributed to long‐term and systemic alleviation of aGvHD symptoms, it is worth investigating whether the beneficial effects of *A. muciniphila* on aGvHD extend to chronic GvHD.

Unlike many idiopathic or spontaneously occurring inflammatory diseases, aGvHD occurs exclusively in patients undergoing HSCT.^[^ ^35^, ^58^ ^]^ This distinct etiological feature allows for preventive strategies for aGvHD despite the complexity of the disease. Our study demonstrates that pre‐administration of *A. muciniphila* prior to HSCT can prevent life‐threatening aGvHD events, highlighting its translational potential as a preventive approach for HSCT recipients.

## 4 Conclusion

This study demonstrates that *A. muciniphila* significantly mitigates aGvHD by preserving intestinal barrier integrity, alleviating dysbiosis, and restoring protective metabolites such as TUDCA. These effects were accompanied by reduced inflammatory responses and modulation of disease‐related transcriptome and microRNA pathways, including suppression of the pro‐inflammatory regulator miR‐155. Both live and pasteurized forms of *A. muciniphila* attenuated disease severity, with live bacteria conferring the strongest protection.

Our findings highlight *A. muciniphila* as a promising microbiome‐based strategy for preventing and treating aGvHD. By simultaneously reinforcing epithelial defenses, restoring microbial and metabolic homeostasis, and rebalancing immune regulation, *A. muciniphila* addresses key drivers of disease progression. Importantly, the efficacy of the pasteurized form supports its translational potential for clinical application in immunocompromised patients. These results provide a strong rationale for advancing *A. muciniphila* toward clinical evaluation as a safe and effective adjunct therapy to improve outcomes in patients undergoing allogeneic stem cell transplantation.

## 5 Experimental Section

### Mice

All animal experiments were approved by the Institutional Animal Care and Use Committee of Kyung Hee University (approval no. KHSASP‐22‐541; see Ethics approval section). Specific‐pathogen‐free BALB/c (H‐2^d^) and C57BL/6 (H‐2^b^) female mice aged 8–10 weeks (n = 6–12, Taconic Biosciences, U.S.A.) were maintained in ventilated plastic isolators (25–26 °C temperature, 48 ± 6% relative humidity and a 12‐h light/12‐h dark cycle) and received sterilized food (Teklad Certified Irradiated. Global 18% Protein Rodent Diet 2918C; ENVIGO, U.S.A.) and water ad libitum; 8–10 week‐old C57BL/6 (H‐2^b^) female mice were used as donors.

### Bone Marrow Transplantation

Following an established protocol^[^ ^59^ ^]^ with some modifications, T cell‐depleted bone marrow cells (5 × 10^6^) with purified T cells (1 × 10^6^) obtained from B6 mice were transferred to lethally irradiated (7 Gy; Best Theratronics, GammaBeam™ 100–80, U.S.A.) BALB/c recipient mice. An analogous procedure was repeated with a reduced cell count (5 × 10^6^ / 0.75 × 10^6^) to establish a murine model of low‐mortality aGvHD. For non‐GvHD controls, a syngeneic bone marrow transplant (i.e., strain‐ and sex‐matched recipients) was performed.

### Preparation of Live and Pasteurized *A. Muciniphila*

The type strain of *A. muciniphila* (*A. muciniphila* Muc^T^, CIP107 961) was cultivated on brain‐heart infusion medium (241830; BD Difco, U.S.A.) supplemented with porcine stomach mucin (0.25%, Type III, Sigma‐Aldrich, U.S.A.), under anaerobic conditions at 37 °C. Bacterial cells harvested during the late exponential growth phase were resuspended in PBS containing sodium thioglycolate (0.005%, Sigma‐Aldrich, U.S.A.). This suspension (4.0 × 10^8^ colony‐forming units per 200 µL) was administered orally to the murine subjects. To prepare pasteurized *A. muciniphila*, the suspension was pasteurized for 30 min at 70 °C, and no viable bacteria were recovered in subsequent cultures.

Three administration regimens were employed according to experimental purpose.

- For prophylactic and longitudinal analyses, mice received daily oral gavage of PBS, live, or pasteurized *A. muciniphila* from 1 month before HSCT until disease onset, with treatment resumed 1 week after onset.
- For irradiation preconditioning experiments, *A. muciniphila* was administered daily for 30 days prior to total‐body irradiation.
- For therapeutic analyses, daily oral gavage was initiated 3 days after aGvHD onset and continued until study termination.

The daily administration regimen was chosen to maintain stable intestinal colonization and promote mucosal adaptation across these experimental settings, consistent with previous studies demonstrating that sustained exposure is required for *A. muciniphila* efficacy.^[^ ^22^, ^38^, ^57^, ^60^, ^61^ ^]^

### Clinical GvHD Scoring

Recipient mice were monitored every two days for clinical indicators of aGvHD including weight loss, hunched posture, reduced activity, changes in fur texture, diarrhea, eye condition, and skin rash. Each observed symptom was assigned a severity score on a scale of 0–2. The scores for each part were summed and used as the disease score for each individual.^[^ ^62^ ^]^

### Histology Analysis

Mice were euthanized 4 weeks after HSCT, and their colon, small intestine, eyes, tongue, and lung tissues were preserved in formaldehyde solution (4%). Fully fixed organs were sectioned to a suitable size for tissue sample preparation at a thickness of ≈2–3 mm. The sectioned specimens were placed in cassettes, labeled with the corresponding specimen number, and subjected to tissue processing for 13 h. These specimens were sectioned into thin slices of ≈3 µm in thickness, adhered to slides, and dried. Sections were deparaffinized and hydrated, followed by rinsing with distilled water. Finally, sections were stained with hematoxylin and eosin (H&E) and Alcian Blue with PAS.

### Gut Permeability Assay

Before the experiment, mice were fasted for 6 h and transferred to sterile cages without feeding or bedding. A FITC‐dextran (4kDa, Sigma‐Aldrich, U.S.A.) was administered by oral gavage with FITC‐dextran (150 µL of 80 mg/mL). 4 h post‐gavage, serum was harvested from peripheral blood and subjected to a 1:1 dilution with PBS. Diluted serum was analyzed using a microplate reader (Synergy Mx Microplate Reader; BioTek, U.S.A.) at excitation and emission wavelengths of 485 and 535 nm, respectively.

### Microbial DNA Isolation and 16s rRNA Gene‐Based Amplicon Sequencing

Fecal samples were collected 3 weeks after HSCT. Metagenomic DNA was extracted from equal amounts of each sample using the QIAamp DNA Stool Mini Kit (51604; Qiagen, Germany) according to the manufacturer's instructions. The hypervariable regions V3–V4 of the 16S rRNA gene were targeted using PCR to generate sequencing amplicons and were amplified with universal bacterial primers 341F (5ʹ‐CCTACGGGNGGCWGCAG‐3ʹ) and 805R (5ʹ‐GACTACHVGGGTATCTAATCC‐3ʹ) using a C1000 thermal cycler (Bio‐Rad, U.S.A.). Triplicate PCR reactions for each DNA template were pooled and purified using a QIAquick PCR Purification Kit (28106; Qiagen, Germany). To prepare for next‐generation sequencing, an index PCR was performed on purified DNA templates to add Illumina sequencing adaptors. The concentration of the purified amplicons was measured using a PicoGreen assay and sequencing was performed on an Illumina MiSeq platform by Macrogen Inc. (Republic of Korea).

### Microbiome Sequencing Data Analyses

The sequencing results were analyzed using the Quantitative Insights Into Microbial Ecology 2 (QIIME2) platform (version 2023.5).^[^ ^63^ ^]^ The sequence data were quality‐filtered and demultiplexed. Using the DADA2 algorithm,^[^ ^64^ ^]^ they were denoised and truncated based on the quality plots. Amplicon sequence variants (ASVs) found in three or fewer samples were removed, and the remaining data were rarefied to match the sample with the lowest read count, normalizing the sequencing depth across all samples. Taxonomic classification of the high‐quality ASVs was then performed using the ‘classify‐sklearn’ feature in the QIIME toolkit, with a pre‐trained Naïve Bayes classifier referencing the SILVA database (version 138).^[^ ^65^ ^]^ Several indices were used to assess the diversity of the microbial community: 1) the observed ASV counts, counting the unique ASVs in each sample to estimate species richness; 2) the Shannon diversity index to measure species abundance and evenness; and 3) Faith's phylogenetic diversity (PD) to measure biodiversity based on phylogenetic relationships within the community. The compositional structure of the microbial community was analyzed using PCoA with an unweighted UniFrac distance. In addition, the PICRUSt2 pipeline (version 2.5.2)^[^ ^66^ ^]^ was used to predict bacterial functional pathways. Human microbiota data (SRA: SRP357021 and SRP322833) from the NCBI SRA database (https://www.ncbi.nlm.nih.gov/sra) were used for gut microbiota analysis in patients with aGvHD.

### RNA Sequencing

Total RNA was extracted from intestinal tissue samples, and its quality was assessed using capillary electrophoresis. Due to partial RNA degradation, library preparation was performed using the SureSelect RNA Direct Mouse Kit (Agilent Technologies, U.S.A.) according to the manufacturer's instructions, instead of the TruSeq stranded mRNA kit originally considered. A total of 16 libraries were constructed and sequenced on an Illumina platform (NovaSeq, paired‐end 101 bp reads, Macrogen Inc, Republic of Korea). The average sequencing output per sample was ≈9–11 Gbp, with a mean Q30 score of ∼96% and an average GC content of 50%, indicating high‐quality reads. Raw sequencing data were provided in FASTQ format and used for downstream differential expression and functional enrichment analyses.

### Small RNA‐Seq and miRNA Analysis

Colon tissues were collected from mice, and total RNA was extracted using the miRNeasy Mini Kit (Qiagen, Germany) according to the manufacturer's instructions. RNA integrity was confirmed with a Bioanalyzer 2100 (Agilent Technologies, U.S.A.). Small RNA libraries were prepared using the NEXTflex Small RNA‐Seq Kit v4 (PerkinElmer, U.S.A.), and sequencing was performed on an Illumina NovaSeq 6000 platform, yielding ≈33–45 million reads per sample. Adapter trimming (3′ adapter: TGGAATTCTCGGGTGCCAAGG) and quality filtering were performed on the raw sequencing data using fastp (v0.23.2),^[^ ^67^ ^]^ followed by adapter removal with cutadapt (v2.6).^[^ ^68^ ^]^ RNA‐seq reads were quantified using Salmon (v1.10.0),^[^ ^69^ ^]^ which performs lightweight pseudo‐alignment and bias‐aware transcript quantification. The Mus musculus reference transcriptome (GENCODE release M37, GRCm39) was downloaded from the GENCODE database (https://www.gencodegenes.org/mouse/).^[^ ^70^ ^]^ A Salmon index was built once from the reference transcriptome FASTA file, and paired‐end trimmed reads were quantified using Salmon with default parameters. For each sample, Salmon generated transcript‐level expression estimates. Post‐quantification quality control was performed using MultiQC (v1.27),^[^ ^71^ ^]^ which summarized mapping rates, fragment length distributions, and overall quantification metrics derived from the Salmon output files.

Transcript‐level quantification files generated by Salmon were summarized to gene‐level counts using the ‘tximport’ package (v1.34.0)^[^ ^72^ ^]^ on R program (v4.4.1).^[^ ^73^ ^]^ Transcript‐to‐gene mapping was derived from the GENCODE annotation file (release M37, GRCm39). Differential expression analysis was performed using the ‘DESeq2’ package (v1.46.0).^[^ ^74^ ^]^ Lowly expressed genes (fewer than 10 counts in at least two samples) were filtered out prior to modeling. DESeq2 was run once to estimate size factors, dispersions, and coefficients across all conditions, followed by pairwise contrasts between experimental groups. Log2 fold changes were further moderated using the ‘apeglm’ shrinkage method.^[^ ^75^ ^]^ Genes with an adjusted *p*‐value (FDR) ≤ 0.05 and |log_2_FC| > 1 were considered significantly differentially expressed.

Post‐analysis gene annotation was performed using ‘AnnotationDbi’^[^ ^76^ ^]^ and ‘org.Mm.eg.db’^[^ ^77^ ^]^ for mouse gene symbols and Entrez IDs, ‘biomaRt’ for Gene Ontology terms,^[^ ^78^ ^]^ and ‘KEGGREST’ for KEGG pathway information.^[^ ^79^ ^]^ Volcano, MA, PCA plots and heatmaps were generated from variance‐stabilized counts using ‘ggplot2’^[^ ^80^ ^]^ and ‘pheatmap’.^[^ ^81^ ^]^ Pathway activity was estimated via ‘ssGSEA’,^[^ ^82^ ^]^ and enrichment analysis was conducted with ‘clusterProfiler’.^[^ ^83^ ^]^ KEGG over‐representation analysis (ORA) for up‐ and down‐regulated DEGs was performed with biomaRt‐based ID conversion and org.Mm.eg.db annotation. Representative pathways such as “Hematopoietic cell lineage” and “Graft‐versus‐host disease” were further visualized as gene–term networks using enrichplot,^[^ ^84^ ^]^ highlighting the DEGs contributing to each pathway.

### Metabolomic Analysis

Each fecal and serum sample (serum samples were collected from peripheral blood and stored at ‐80 °C) were divided into equal aliquots and suspended in a solution (acetonitrile:methanol:distilled water = 1:1:2) with formic acid (0.1%) and an internal standard (Reserpine, Sigma‐Aldrich, U.S.A.). The suspended aliquots were homogenized using a vortex and centrifuged at 16,422 × *g* for 10 min at 4 °C to isolate the supernatant, which was filtered via polytetrafluoroethylene syringe filters (0.22 µm). After sample preparation, untargeted and targeted metabolomic analyses were performed using liquid chromatography‐quadrupole time‐of‐flight mass spectrometry (LC‐Q‐TOF‐MS). The system incorporated a 1290 Infinity high‐efficiency liquid chromatography unit (UHPLC Infinity 1290, Agilent Tech., U.S.A.) paired with a Q‐TOF mass spectrometer (6550 iFunnel Q‐TOF‐MS, Agilent Technologies, U.S.A.). An Agilent Eclipse Plus C18 column (2.1 mm x 100 mm, 2.1 µm) was used for both analyses. Output data from untargeted metabolomic analysis were clustered via principal component analysis using Metaboanalyst 6.0 workflow.^[^ ^85^ ^]^ Targeted metabolomic analysis of bile acids was conducted according to the protocol reported by Baek et al.^[^ ^86^ ^]^ with slight modifications. The bile acids were quantified using certified reference compounds (Table S1, Supporting Information).

### Flow Cytometry Analysis

PBMCs were extracted from the mice. Each single‐cell suspension was adjusted to a cell count of 1 million, followed by incubation with antibodies, including the Fc Block (BD Pharmingen, U.S.A.), for 20 min at 4 °C. Subsequently, the cells were washed with staining buffer composed of PBS enriched with fetal bovine serum (1%). Flow cytometry analysis was conducted using the BD FACSCanto II (BD Biosciences, U.S.A.). The fluorochrome‐conjugated monoclonal antibodies and solutions for intracellular staining used for flow cytometry analysis are listed in Table S2 (Supporting Information).

### Proteome Profiler Mouse Cytokine Array

The relative expression of cytokines and chemokines in murine serum was quantified using the Proteome Profiler Mouse Cytokine Array Kit, Panel A (ARY006; R&D Systems, U.S.A.), following the manufacturer's instructions.

### Complete Blood Count Analysis

The blood samples were obtained using tubes pre‐filled with EDTA and analyzed using a hematology analyzer (BC‐5000 VET; MINDRAY, CHINA) within 24 h post collection.

### Colony‐Forming Unit (CFU) Assay

Colony‐forming assays were performed according to the manufacturer's instructions (# 04445, STEMCELL Technologies) with minor modifications. Human CD34⁺ hematopoietic stem/progenitor cells (CBP3400.5C, CGT Global, U.S.A.) were co‐cultured with HT‐29 intestinal epithelial cells in a transwell system and exposed to TNF‐α, IFN‐γ (20 ng/mL each), and LPS (1 µg/mL) for 24 h. After stimulation, CD34⁺ cells were harvested and plated in MethoCult™ (H4435, Stemcell Technologies, Canada) medium (500 cells/plate, 35 mm) and incubated for 14 days at 37 °C in 5% CO_2_. Colonies were counted under an inverted microscope and classified as BFU‐E, CFU‐G/M/GM, or CFU‐GEMM based on standard morphological criteria.

### Statistical Analysis

Permutational multivariate analysis of variance (PERMANOVA) was performed on QIIME2. In addition, the Kruskal–Wallis test followed by Dunn's multiple comparisons test was performed using the ‘dunn.test’ package^[^ ^87^ ^]^ in R program^[^ ^73^ ^]^ to identify statistically significant differences among multiple groups, while pairwise comparisons were analyzed using the Wilcoxon rank‐sum test implemented in the ‘stats’ package.^[^ ^73^ ^]^ Moreover, survival analysis with cox proportional hazards model was conducted to show the survival conditions of each group during the experiment periods and the significant difference of survival rate between groups using ‘survival’ package^[^ ^88^ ^]^ on R.

### Ethics Approval

All animal studies were performed in accordance with guidelines approved by the Institutional Animal Care and Use Committee of Kyung Hee University (KHSASP‐22‐541).

## Conflict of Interest

The authors declare no conflict of interest.

## Author Contributions

J.‐E. Han designed the experiment scheme, performed all experiments, and data analysis, and wrote the manuscript. D.‐S. Lee collaborated with data analysis, data visualization and reviewed the manuscript. S.‐W. Jeong collaborated on conducting the experiments. J.‐H. Yun contributed to review of the manuscript. S. Kang contributed to writing the manuscript. S. Jang and E. Lee collaborated on the in vitro experiments. J. H. Baek and C. O. Jeon collaborated on the metabolomic analysis. J.‐W. Bae supervised this work.

## Supporting information



Supporting Information

## Data Availability

Data are available upon reasonable request. The 16s rRNA sequencing data of the intestinal microbiota of mice obtained through this study can be found in the NCBI database (https://www.ncbi.nlm.nih.gov; BioProject: PRJNA1154330).
