# Supplementary material for: Pre‐Administration of Akkermansia Muciniphila Prevents the Development of Severe Acute Graft‐Versus‐Host Disease in Systemic Organs
Source: Adv Sci (Weinh). 2025 Nov 30;13(9):e15982. doi: 10.1002/advs.202415982 (PMC12903985; doi:10.1002/advs.202415982)
Supplement: Supplementary file 1 — Supporting Information [file ADVS-13-e15982-s001.docx]

Supporting Information

**Material and Methods**

**Total-body irradiation (TBI)–induced intestinal injury**

BALB/c mice were lethally irradiated (7 Gy; Best Theratronics, GammaBeam™ 100-80, U.S.A.). Mice were orally administered live or pasteurized *A. muciniphila* (4.0 × 10^8 CFU/200 μL PBS) once daily 30 days prior to irradiation. PBS-treated mice were included as controls. Intestinal tissues were collected 3 days after TBI for histological analysis (H&E). Diarrhea incidence was evaluated on day 3 after irradiation using a predefined scoring scale.

**Human PBMC–HT-29 cells co-culture assays**

The overall workflow was adapted from a published protocol with modifications [1]. Human PBMCs (PBMNC050C, CGT Global, U.S.A.) were cultured in RPMI 1640 medium supplemented with 10% heat-inactivated FBS, 2 mM L-alanyl-glutamine, and 1% penicillin–streptomycin at 37 °C in 5% CO₂. Cells were seeded at 1 × 10⁶ cells/mL and stimulated with LPS (1 µg/mL; L2630, Sigma-Aldrich, U.S.A.) and anti-CD3/CD28 antibodies (clones OKT3/CD28.2, BioLegend, U.S.A.) for 24 h to generate cytokine-producing inflammatory PBMCs for co-culture experiments. HT-29 cells (30038, Korean Cell Line Bank, Republic of Korea) were maintained in DMEM containing 10% FBS and seeded onto transwell inserts (0.4 µm pore size; Corning) until confluent monolayers were established. Inflammatory PBMCs were placed in the basolateral chamber, whereas pasteurized *A. muciniphila* (multiplicity of infection, MOI = 1), tauroursodeoxycholic acid (TUDCA; 250 µM; T0266, Sigma-Aldrich, U.S.A.), or the TGR5 antagonist SBI-115 (100 µM; HY-112865, MedChemExpress, U.S.A.) were applied to the apical chamber. After 24 h, barrier integrity was assessed by measuring apical-to-basolateral flux of FITC–dextran (4 kDa; 46944, Sigma-Aldrich, U.S.A.) using a Spark 10M plate reader (Tecan, Switzerland) at excitation/emission wavelengths of 485/535 nm. Cell viability was determined by MTT assay (M2128, Sigma-Aldrich, U.S.A.). Cells were incubated with MTT reagent for 4 h at 37 °C, formazan crystals were solubilized in DMSO, and absorbance was measured at 560 nm.

**miR-155 mimic transfection**

For miR-155 mimic transfection, HT-29 cells were transfected with either hsa-miR-155-5p mimic (20 nmol; HY-R00316, MedChemExpress, U.S.A.) or a negative control mimic (HY-004474, MedChemExpress, U.S.A.) using Lipofectamine RNAiMAX (13778075, Thermo Fisher Scientific, U.S.A.) in Opti-MEM medium, following the manufacturer’s protocol.

**Fecal protein–bacteria interaction assays**

Fecal proteins were extracted from mouse fecal samples as previously described [2]. Briefly, mouse fecal pellets were weighed and homogenized in PBS. Homogenates were centrifuged at 15,000 × g for 20 min at 4 °C, and the supernatants were filtered through 0.22 µm syringe filters. Protein concentrations were measured using the BCA protein assay (A55865, Thermo Fisher Scientific, U.S.A.), and protein solutions were adjusted to the desired concentrations for co-culture.

*Escherichia coli* and *Enterococcus faecium* strains were isolated directly from mouse fecal samples by streaking onto BHI agar under anaerobic conditions. Colonies were selected, grown overnight in BHI broth at 37 °C under anaerobic conditions, harvested by centrifugation (10,000 × g, 5 min), washed twice in sterile PBS, and resuspended to an Optical Density (OD) of 0.5 0.5 in PBS.

Under anaerobic conditions, equal volumes of fecal protein extract and bacterial suspension were mixed and incubated at 37 °C for 24 h. Colony-forming units (CFU) were counted and CFU/mL calculated by multiplying by the dilution factor.

**ELISA (mouse REG3G and LYZ1)**

Samples were prepared on ice in PBS containing protease inhibitors and clarified by centrifugation (≥10,000 ×g, 10 min, 4 °C). Supernatants were stored at −80 °C until analysis and thawed once. Mouse REG3G concentrations were measured using REG3G ELISA Kit (OKCD00733-96W, Aviva Systems Biology, U.S.A.), and mouse lysozyme was measured using LYZ1 ELISA Kit (OKCD00269-96W, Aviva Systems Biology, U.S.A.), according to the manufacturers’ instructions. Standards and samples were run in duplicate with appropriate dilution factors. Plates were read at 450 nm on a Spark 10M plate reader (Tecan).

**Fecal metabolite extraction**

For metabolite extraction, fecal samples (0.04 g stored at -80 °C) were prepared for targeted metabolomics of short-chain fatty acids (SCFAs) using gas chromatography-mass spectrometry (GC-MS). Samples were dissolved in an extraction solution (0.5% phosphoric acid: 500 μM 4-methyl valeric acid in butanol=1:1) and homogenized by vortexing for 1 min. The mixture was then centrifuged at 16,422 × *g* for 10 min at 4 °C, and the supernatants were passed through a polytetrafluoroethylene (0.2 μm) filter (BS20-PO13, BioFACT, Republic of Korea).

**Fecal short-chain fatty acids analysis**

Targeted metabolomics analysis of SCFAs was performed using an Agilent 7820A GC-MS system (Agilent Technologies, U.S.A.). Chromatographic separation was achieved using a DB-Wax column (50 m × 200 μm × 0.20 μm, Agilent Technologies, U.S.A.). SCFA quantification was performed with certified reference compounds (Table S1).

**Table S1.** Reference compounds used for metabolomic analysis.

| Compound | Use | Source |
| --- | --- | --- |
| Chenodeoxycholic acid | LC-MS | Sigma-Aldrich |
| Deoxycholic acid | LC-MS | TGI |
| Lithocholic acid | LC-MS | Sigma-Aldrich |
| Tauroursodeoxycholic Acid | LC-MS | Sigma-Aldrich |
| Ursodeoxycholic acid | LC-MS | TGI |
| Glycocholic acid | LC-MS | Sigma-Aldrich |
| Acetic acid | GC-MS | Sigma-Aldrich |
| Butyric acid | GC-MS | Sigma-Aldrich |
| Propionic acid | GC-MS | Sigma-Aldrich |

**Table S2.** Antibodies and solutions for intracellular staining.

| Compound | Use | Source |
| --- | --- | --- |
| CD45–Pacific Blue | Flow Cytometry | BioLegend |
| CD3-AmCyan | Flow Cytometry | BioLegend |
| CD8-FITC | Flow Cytometry | BioLegend |
| CD4- PE (phycoerythrin)/Cy7 | Flow Cytometry | BioLegend |
| CD44–APC | Flow Cytometry | BioLegend |
| IFN-γ–PE | Flow Cytometry | BioLegend |
| Foxp3-PE | Flow Cytometry | BioLegend |
| Cytofix/Cytoperm solution | Flow Cytometry | BD Bioscience |
| Foxp3/Transcription-Factor Staining Buffer Set | Flow Cytometry | eBioscience |

**
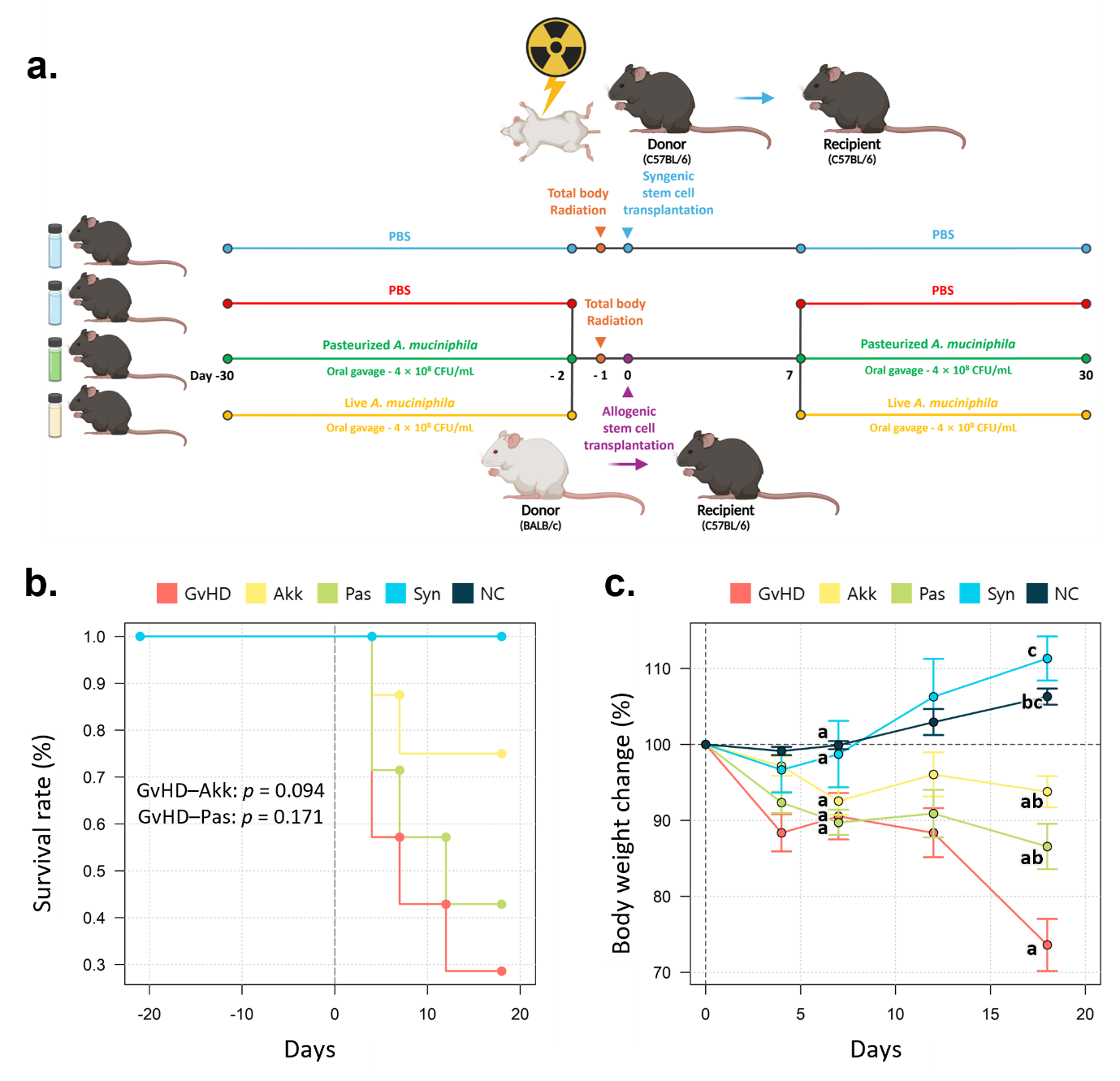
**

**Figure S1. Ameliorating effects of *A. muciniphila* in a donor-recipient switched model.** (a) Experimental design for the donor-recipient switched model. Recipients were monitored for survival rate (b) and body weight change (c) until 18 days post-HSCT (n = 5–8). A survival analysis using the Cox proportional hazards model confirmed the difference in survival between the Akk and Pas groups for GvHD group (b). The statistical analysis using Kruskal–Wallis test with Dunn's multiple comparisons test was conducted to identify statistical differences between groups (c). Different letters on the figures indicate statistically significant differences (*p* < 0.05); error bars represent the standard error of the mean. (Mice group abbreviation: GvHD, PBS-administered aGvHD group; Akk, aGvHD group administered with live *A. muciniphila*; Pas, aGvHD group administered with pasteurized *A. muciniphila*; Syn, PBS-administered syngeneic-HSCT group; NC, normal control mice administered only PBS)


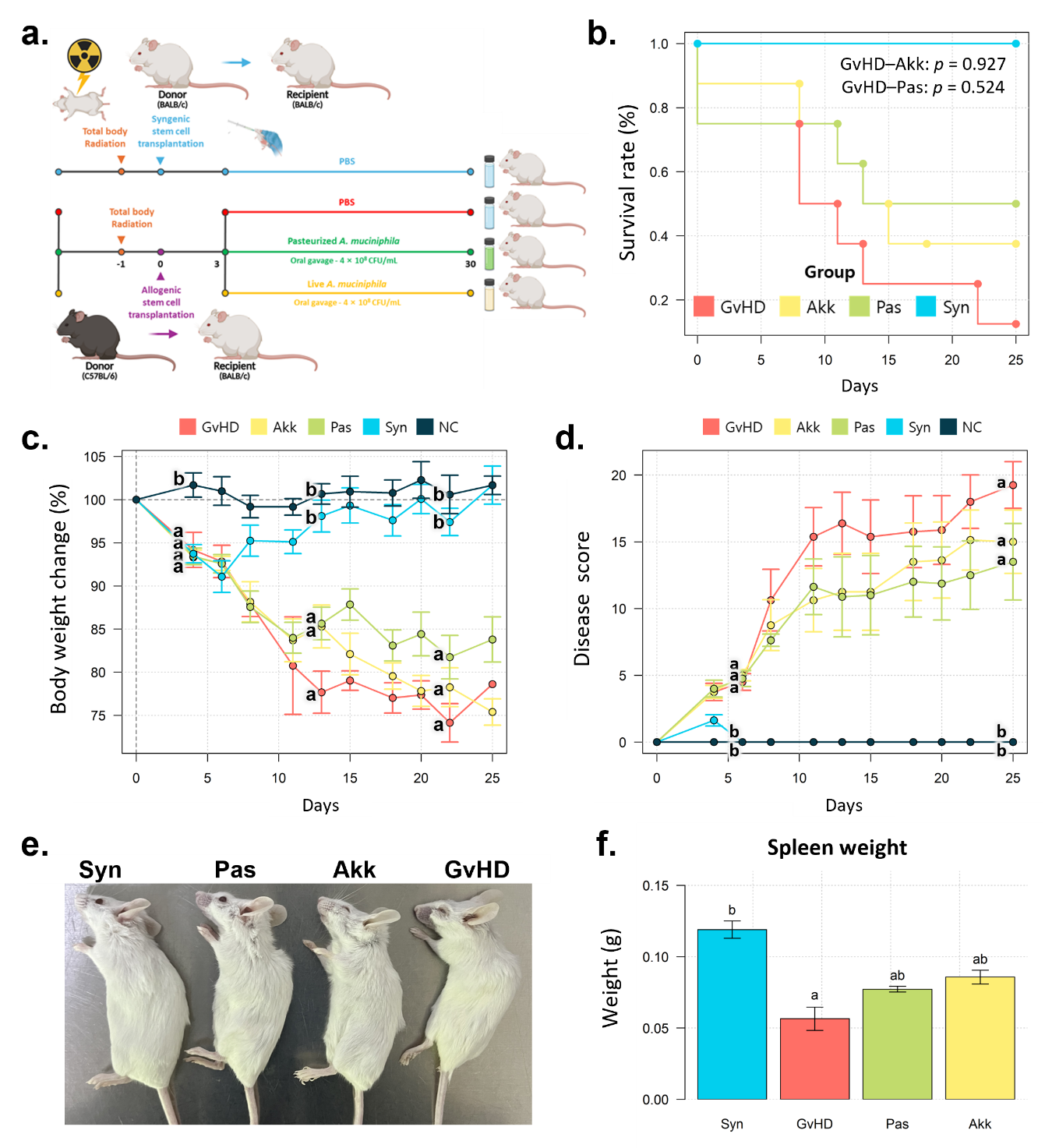


**Figure S2. Post-administration effects of *A. muciniphila* after aGvHD onset.** (a) Schematic representation of the experimental design. After allogeneic hematopoietic stem cell transplantation (HSCT), mice were randomly assigned into five groups: GvHD, PBS-administered aGvHD group; Akk, aGvHD group treated with live *A. muciniphila*; Pas, aGvHD group treated with pasteurized *A. muciniphila*; Syn, PBS-administered syngeneic-HSCT group; and NC, normal control mice administered PBS only. Pasteurized or live *A. muciniphila* (4 × 10⁸ CFU/mL) was orally administered daily starting three days after HSCT and continued until day 30. Recipients were monitored for survival rate (b), body weight change (c), and clinical score (d) up to 30 days after HSCT (n = 6–12). Representative pictures of the overall appearance (e). Mice spleens were collected 4 weeks post-HSCT to confirm the weight (f). Survival analysis with Cox proportional hazards model was used to confirm the difference in survival trends between the Akk and Pas groups for GvHD group (b). The Kruskal–Wallis test followed by Dunn’s multiple comparisons test was performed to determine statistical differences between groups (c, d, f). Different letters in the figures indicate statistically significant differences (*p* < 0.05) and error bars indicate the standard error of the mean.


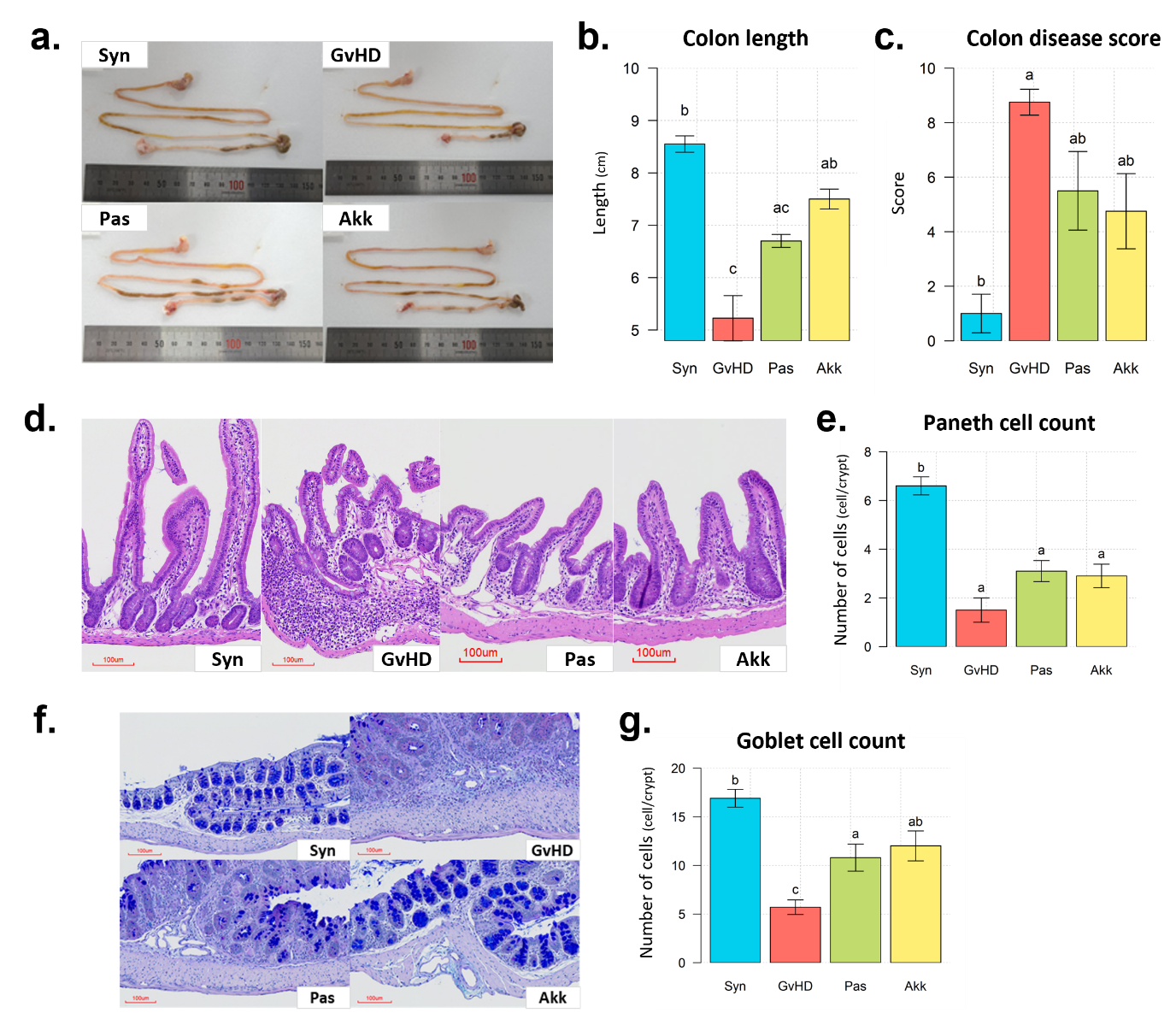


**Figure S3. Administration of *A. muciniphila* after aGvHD onset shows modest improvement in intestinal pathology.** The gastrointestinal (GI) tracts were collected at 3 weeks post-HSCT to assess the severity of intestinal damage (n = 3 per group). Representative phenotype images of the GI tract (a) and colon length measurements (b). Colon disease scores (c). Representative H&E-stained histological images of small-intestinal crypts (d, e) with quantification of Paneth cells per crypt. Representative Alcian blue/PAS-stained colon sections (f, g) with quantification of goblet cells per crypt. The Kruskal–Wallis test followed by Dunn’s multiple comparisons test was performed to determine statistical differences between groups (b, c, e, g). Different letters in the figures indicate statistically significant differences (*p* < 0.05) and error bars indicate the standard error of the mean. (Mice group abbreviation: GvHD, PBS-administered aGvHD group; Akk, aGvHD group administered with live *A. muciniphila*; Pas, aGvHD group administered with pasteurized *A. muciniphila*; Syn, PBS-administered syngeneic-HSCT group)


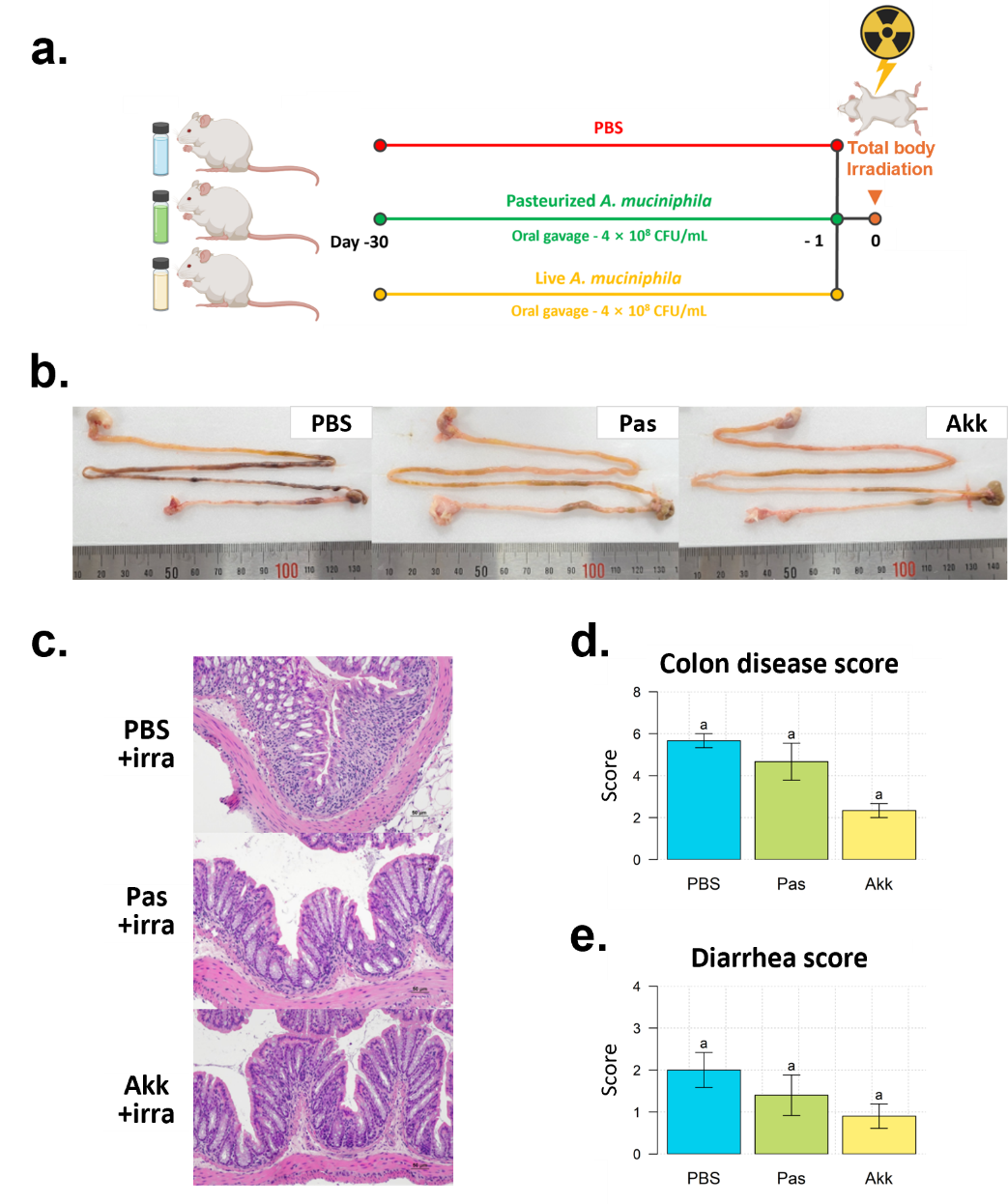


**Figure S4. Radioprotective effects of *A. muciniphila* on irradiation-induced intestinal injury.** Schematic illustration of the total-body irradiation (TBI) experiment (a). Mice were randomly divided into three groups: PBS+irra, PBS-administered TBI group; Akk+irra, TBI group administered with live *A. muciniphila*; and Pas+irra, TBI group administered with pasteurized *A. muciniphila*. Mice were administered PBS, live, or pasteurized *A. muciniphila* daily by oral gavage starting 1 month before TBI. The GI tracts were collected 3 days post-TBI. Representative phenotype images of the GI tract (b) and colons (c). Quantification of colon disease (d) and diarrhea scores (e). The Kruskal–Wallis test followed by Dunn’s multiple comparisons test was performed to determine statistical differences between groups (d, e). Different letters in the figures indicate statistically significant differences (*p* < 0.05) and error bars indicate the standard error of the mean.


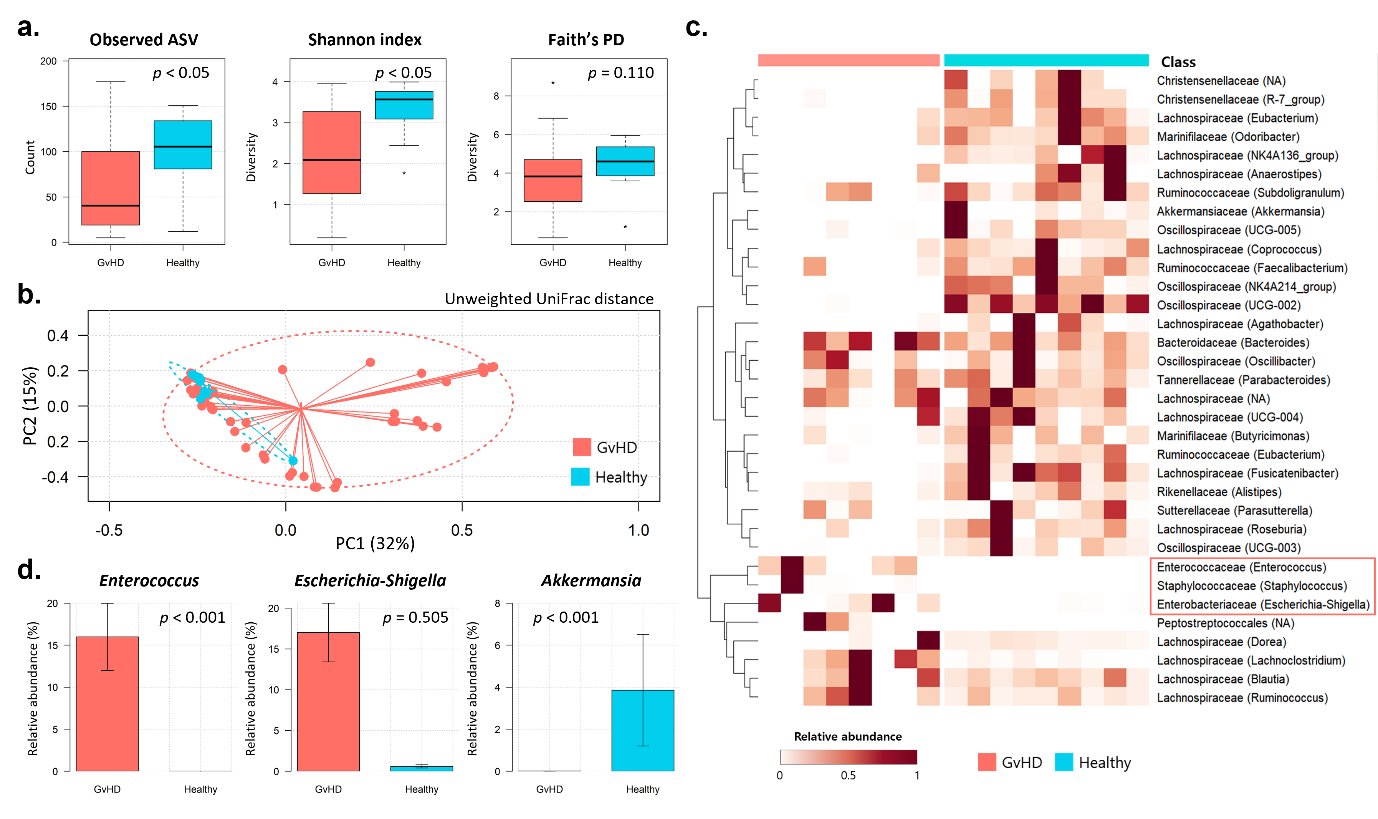


**Figure S5. Analysis of gut microbiota composition in human patients with aGvHD using short read archive (SRA) data.** Analysis of gut microbiota composition in human patients with aGvHD using SRA data from the NCBI database. (a) Alpha diversity comparison of each group was performed using observed ASV, Shannon diversity index, and Faith's Phylogenetic Diversity (PD) index. PCoA plot (b) and heatmap (c) showing the differential abundance of bacterial genera between healthy controls and patients with aGvHD. (d) Relative abundance of the identified genera *Akkermansia*, *Enterococcus*, and *Escherichia**-Shigella* in patients with aGvHD and healthy controls. The statistical analysis using Wilcoxon rank-sum test was conducted to identify statistical differences between two groups (a, d). Error bars represent the standard error of the mean. (Group abbreviation: GvHD, patients with aGvHD; Healthy, healthy human)


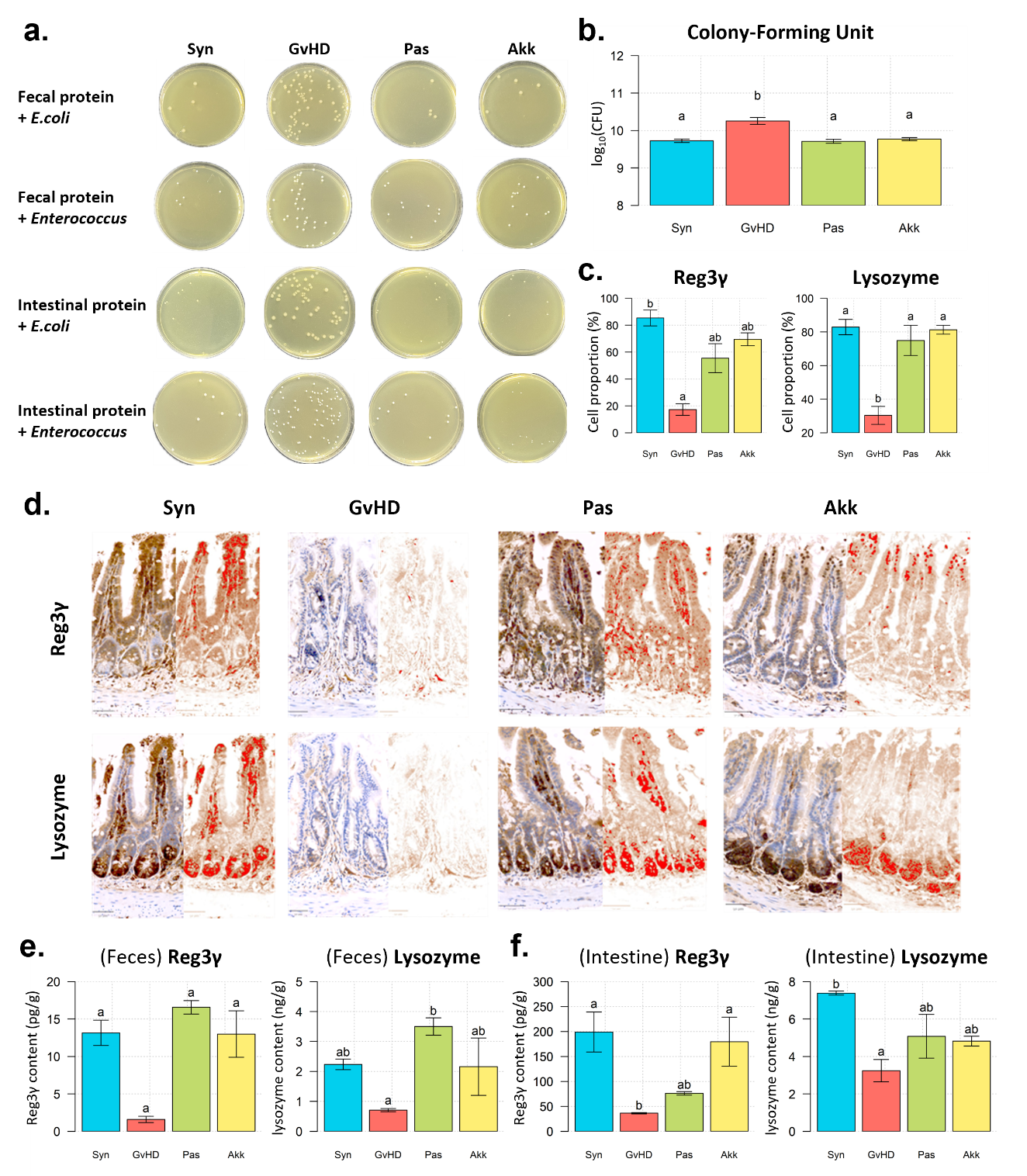


**Figure S6. Suppression of pathogenic bacterial growth and preservation of intestinal antimicrobial defense by *A. muciniphila* treatment**. Fecal and intestinal protein extracts were prepared from the GvHD, Akk, Pas, and Syn groups to evaluate their inhibitory effects on pathogenic bacteria. Representative colony formation of *Enterococcus* and *Escherichia–Shigella* cultured with fecal or intestinal extracts (a), and corresponding viable bacterial counts (b). Quantification of Reg3γ- and lysozyme-positive cells per field (c), and representative immunohistochemistry images showing their expression in intestinal tissues (d). Quantification of Reg3γ and lysozyme levels in fecal samples (e) and intestinal tissues (f) by ELISA. Statistical differences between groups were analyzed using the Kruskal–Wallis test followed by Dunn’s multiple comparisons test. Different letters in the figures indicate statistically significant differences (*p* < 0.05), and error bars represent the standard error of the mean. (Group abbreviations: GvHD, PBS-administered aGvHD group; Akk, aGvHD group treated with live *A. muciniphila*; Pas, aGvHD group treated with pasteurized *A. muciniphila*; Syn, PBS-administered syngeneic HSCT group.)


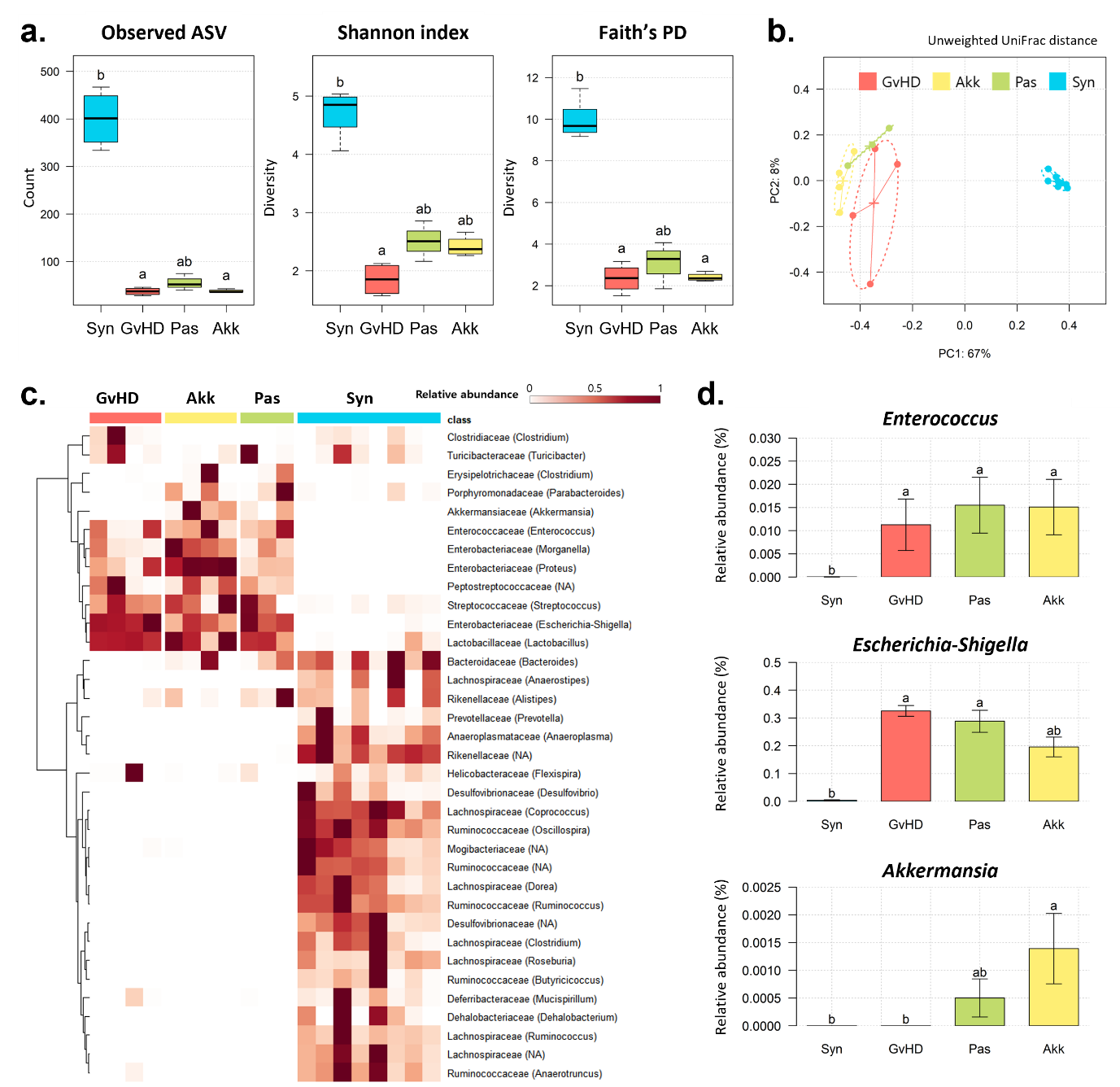


**Figure S7. Limited alterations in gut microbiota composition following administration of *A. muciniphila* after aGvHD onset.** Bacterial 16S rRNA sequencing was performed using fecal samples collected 3 weeks after HSCT (n = 3–4 per group). (a) Alpha diversity comparison of each group was performed using observed ASV, Shannon index, and Faith’s Phylogenetic Diversity (PD) index. (b) Clustering of gut microbiota using PCoA with unweighted UniFrac distances. (c) Heat map showing the relative abundance of bacterial taxa at the genus level. (d) Relative abundance of *Enterococcus*, and *Escherichia–Shigella* and *A. muciniphila* by groups. The Kruskal–Wallis test followed by Dunn’s multiple comparisons test was performed to determine statistical differences between groups (a, d). Different letters in the figures indicate statistically significant differences (*p* < 0.05) and error bars indicate the standard error of the mean. (Mice group abbreviations: GvHD, PBS-administered aGvHD group; Akk, aGvHD group administered with live *A. muciniphila*; Pas, aGvHD group administered with pasteurized *A. muciniphila*; Syn, PBS-administered syngeneic-HSCT group.)


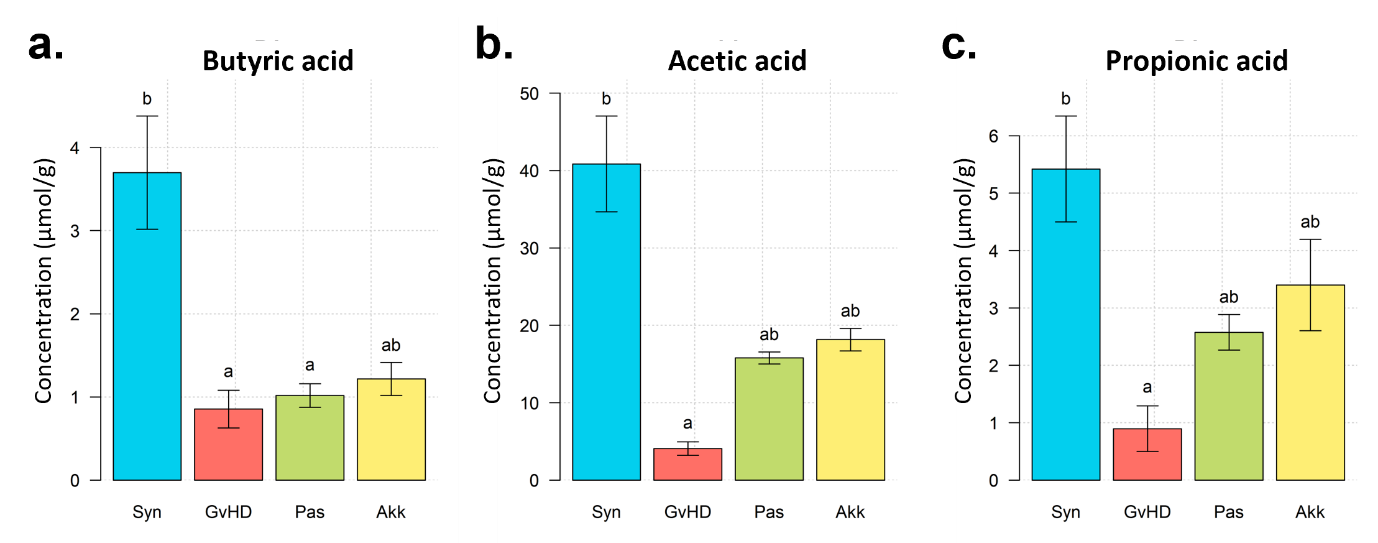


**Figure S8. Targeted metabolomic analysis of short-chain fatty acids.** Targeted metabolomic analysis of fecal short-chain fatty acids (n = 3); (a) Butyric acid, (b) Acetic acid, and (c) Propionic acid. The Kruskal–Wallis test followed by Dunn’s multiple comparisons test was performed to determine statistical differences between groups. Different letters in the figures indicate statistically significant differences (*p* < 0.05) and error bars indicate the standard error of the mean. (Mice group abbreviation: GvHD, PBS-administered aGvHD group; Akk, aGvHD group administered with live *A. muciniphila*; Pas, aGvHD group administered with pasteurized *A. muciniphila*; Syn, PBS-administered syngeneic-HSCT group)


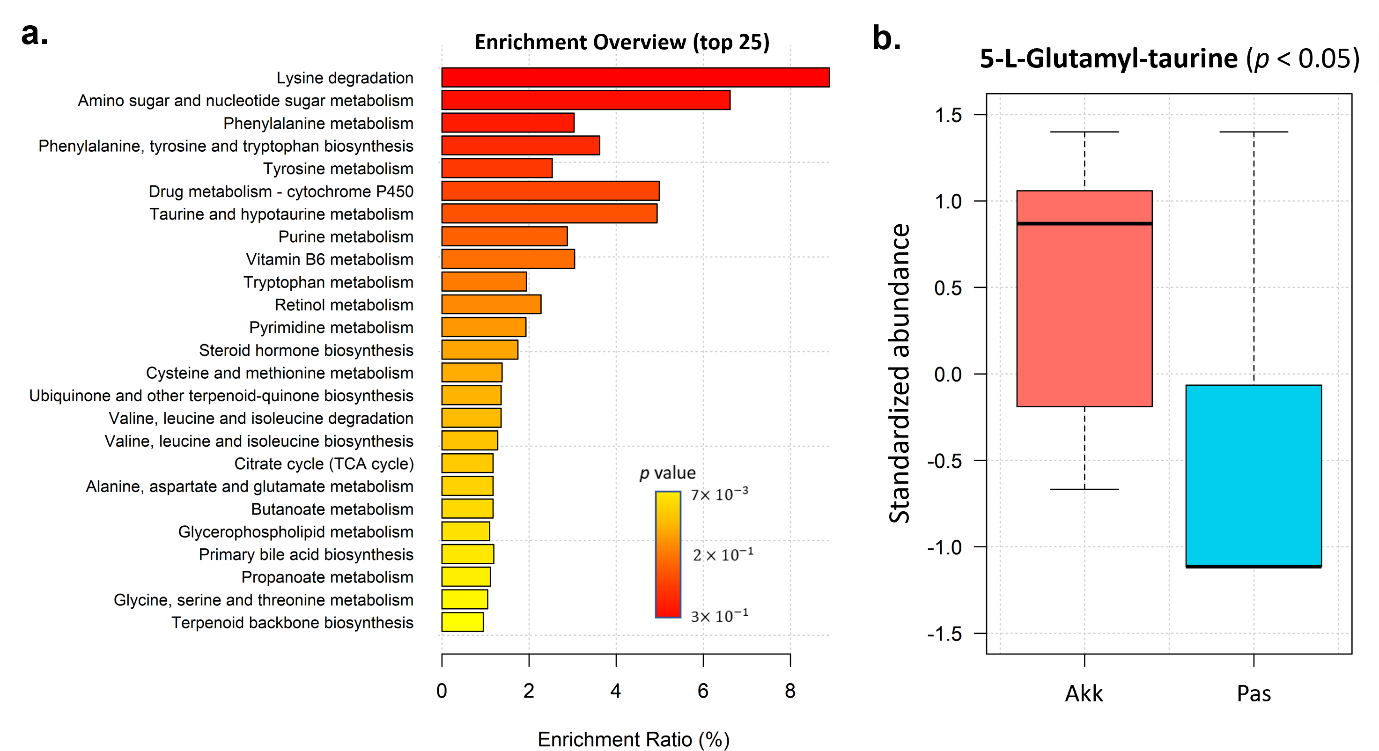


**Figure S9. KEGG pathway enrichment analysis of differentially enriched fecal metabolites between the Akk and Pas groups.** (a) KEGG pathway enrichment overview of the top 25 significantly enriched metabolic pathways identified from differentially enriched fecal metabolites between the Akk and Pas groups. *p*-values for pathway enrichment were calculated using Over-Representation Analysis in the Metabolite Set Enrichment Analysis module of MetaboAnalyst (version 6.0). (b) Standardized abundance of the representative taurine-related metabolite S-L-Glutamyl-taurine. The statistical analysis using Wilcoxon rank-sum test was conducted to identify statistical differences between two groups.


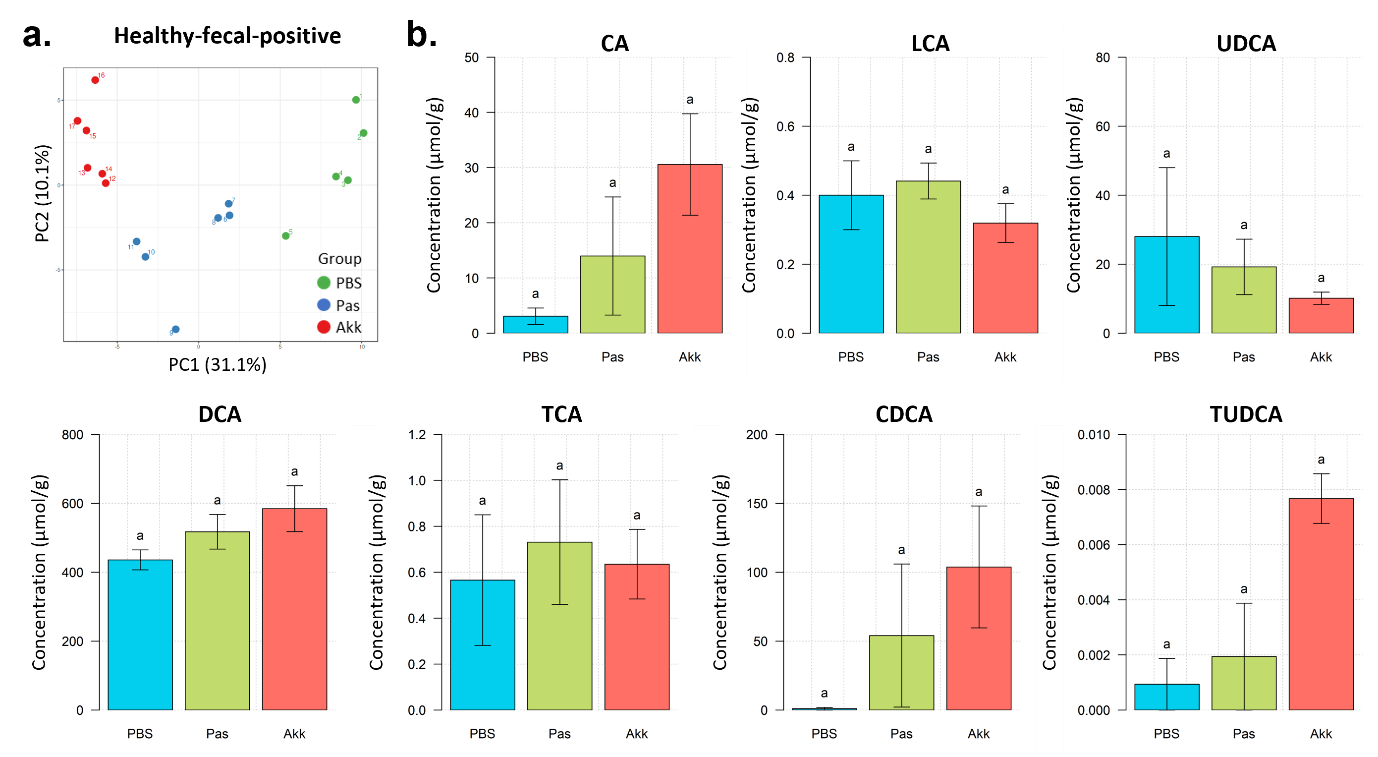


**Figure S10. Metabolomic analyses reveal distinct metabolic shifts and bile acid modulation in mice administered with *A. muciniphila* prior to disease onset.** Mice were orally administered PBS, pasteurized or live *A. muciniphila* (Pas or Akk) daily for 1 month prior to disease induction, and fecal samples were collected for metabolomic analyses. (a) Principal component analysis (PCA) plot showing distinct clustering of fecal metabolite profiles among the three groups. (b) Targeted metabolomic analysis of fecal bile acids, including cholic acid (CA), lithocholic acid (LCA), ursodeoxycholic acid (UDCA), deoxycholic acid (DCA), taurocholic acid (TCA), chenodeoxycholic acid (CDCA), and tauroursodeoxycholic acid (TUDCA). The Kruskal–Wallis test followed by Dunn’s multiple comparisons test was performed to determine statistical differences between groups. Different letters in the figures indicate statistically significant differences (*p* < 0.05) and error bars indicate the standard error of the mean.

**
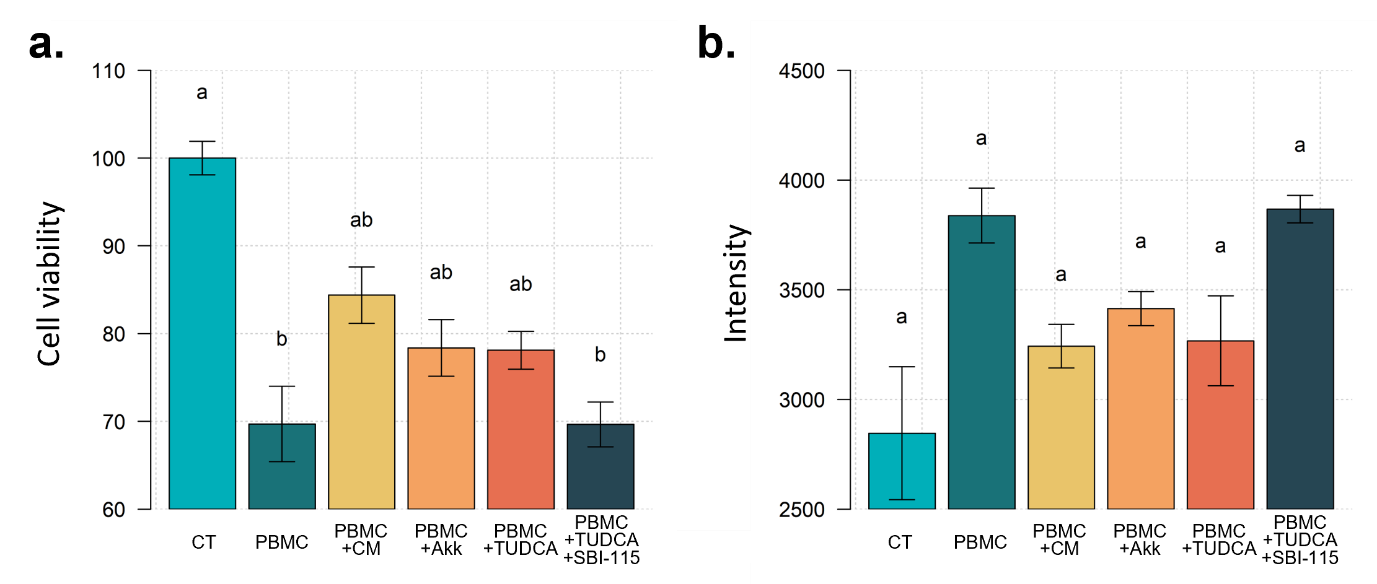
**

**Figure S11. TUDCA protection is blocked by TGR5 antagonist in PBMC–HT-29 co-culture.** Human peripheral blood mononuclear cells (PBMCs) were stimulated with LPS and anti-CD3/CD28 antibodies for 24 h to induce an inflammatory phenotype and then co-cultured with HT-29 epithelial monolayers in a transwell system. Pasteurized *A. muciniphila* (MOI = 1), tauroursodeoxycholic acid (TUDCA; 250 µM), or the TGR5 antagonist SBI-115 (100 µM) were applied to the apical chamber for 24 h. (a) HT-29 cell viability assessed by MTT assay. (b) Epithelial permeability determined by apical-to-basolateral flux of FITC–dextran (4 kDa). The Kruskal–Wallis test followed by Dunn’s multiple comparisons test was performed to determine statistical differences between groups. Different letters in the figures indicate statistically significant differences (*p* < 0.05) and error bars indicate the standard error of the mean. (Abbreviation: CT, PBMC untreated control; CM, *A. muciniphila*-conditioned medium, Akk, Pasteurized *A. muciniphila*; SBI-115, TGR5 antagonist)


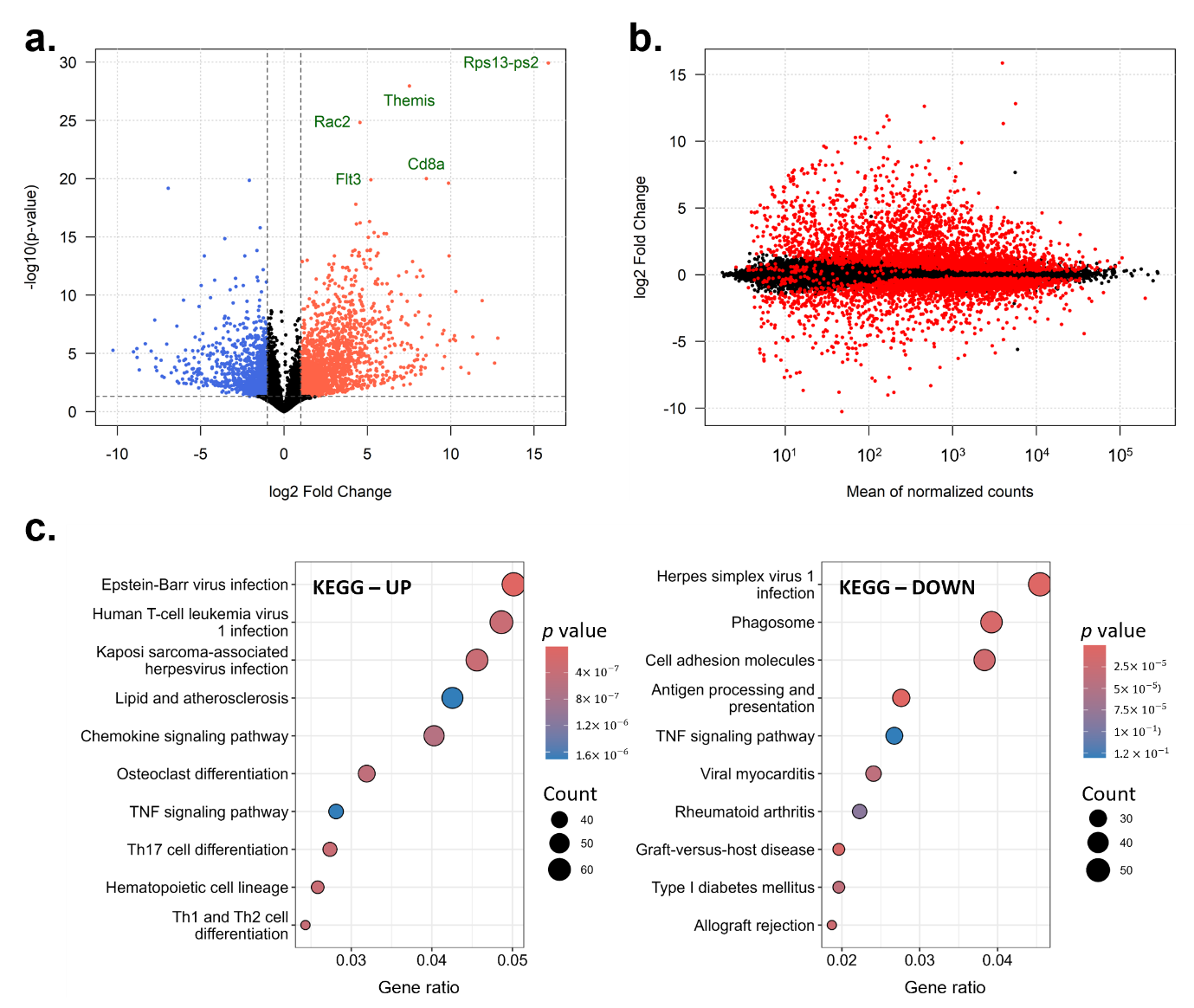


**Figure S12. Transcriptomic comparison reveals distinct immune regulatory pathways between live and pasteurized *A. muciniphila* treatment**. Differential gene expression analysis was performed using RNA-seq data from intestinal tissues of the Akk and Pas groups. (a) Volcano plot showing differentially expressed genes (DEGs) between the Pas and Akk groups. (b) MA plot illustrating the distribution of log₂ fold changes against normalized read counts for each gene. (c) KEGG over-representation analysis of upregulated and downregulated DEGs in the Akk versus Pas comparison. Significance threshold was set at an adjusted *p* < 0.05, and pathway enrichment significance was determined using the Benjamini–Hochberg false discovery rate (FDR) correction.


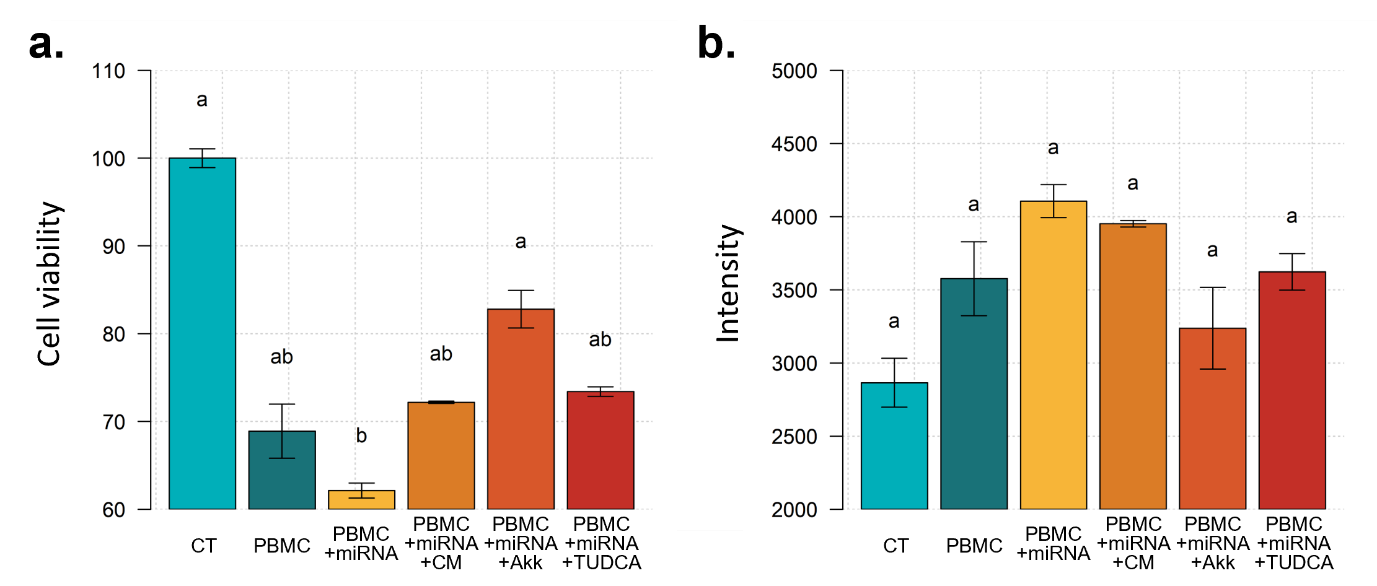


**Figure S13. *A. muciniphila* and TUDCA alleviate miR-155 mimic-induced epithelial injury in PBMC–HT-29 co-culture.** HT-29 cells were transfected with a miR-155 mimic or negative control and co-cultured with inflammatory PBMCs to evaluate epithelial barrier integrity. Cells were treated with pasteurized *A. muciniphila* (MOI = 1) or tauroursodeoxycholic acid (TUDCA; 250 µM) for 24 h. (a) Cell viability measured by MTT assay. (b) Epithelial permeability determined by apical-to-basolateral flux of FITC–dextran (4 kDa). The Kruskal–Wallis test followed by Dunn’s multiple comparisons test was performed to determine statistical differences between groups. Different letters in the figures indicate statistically significant differences (*p* < 0.05) and error bars indicate the standard error of the mean. (Abbreviation: CT, PBMC untreated control; miRNA, miR-155 mimic; CM, *A. muciniphila*-conditioned medium, Akk, Pasteurized *A. muciniphila*)

[1] G. Astre, L. Créchet, N. Pomié, O. Pereira, P. D. Cani, C. Knauf, A. Abot, *STAR protocols* **2024**, *5* (4), 103416.

[2] K. K. Jang, T. Heaney, M. London, Y. Ding, G. Putzel, F. Yeung, D. Ercelen, Y.-H. Chen, J. Axelrad, S. Gurunathan, *Cell Host & Microbe* **2023**, *31* (9), 1450.
